# Exosomal lncRNA PVT1/VEGFA Axis Promotes Colon Cancer Metastasis and Stemness by Downregulation of Tumor Suppressor miR-152-3p

**DOI:** 10.1155/2021/9959807

**Published:** 2021-07-15

**Authors:** Shiue-Wei Lai, Ming-Yao Chen, Oluwaseun Adebayo Bamodu, Ming-Shou Hsieh, Ting-Yi Huang, Chi-Tai Yeh, Wei-Hwa Lee, Yih-Giun Cherng

**Affiliations:** ^1^Division of Hematology-Oncology, Department of Internal Medicine, Tri-Service General Hospital, National Defense Medical Center, Taipei, Taiwan; ^2^Department of Internal Medicine, Tri-Service General Hospital Penghu Branch, Penghu, Taiwan; ^3^Division of Gastroenterology and Hepatology, Department of Internal Medicine, Taipei Medical University-Shuang Ho Hospital, New Taipei City, Taiwan; ^4^Division of Gastroenterology and Hepatology, Department of Internal Medicine, School of Medicine, College of Medicine, Taipei Medical University, Taipei, Taiwan; ^5^Department of Medical Research & Education, Taipei Medical University-Shuang Ho Hospital, New Taipei City 235, Taiwan; ^6^Department of Medical Laboratory Science and Biotechnology, Yuanpei University of Medical Technology, Hsinchu 300, Taiwan; ^7^Department of Pathology, Taipei Medical University-Shuang Ho Hospital, New Taipei City, Taiwan; ^8^Department of Anesthesiology, Shuang ho Hospital, Taipei Medical University, New Taipei City, Taiwan; ^9^Department of Anesthesiology, School of Medicine, College of Medicine, Taipei Medical University, Taipei, Taiwan

## Abstract

**Background:**

Treating advanced colon cancer remains challenging in clinical settings because of the development of drug resistance and distant metastasis. Mechanisms underlying the metastasis of colon cancer are complex and unclear.

**Methods:**

Computational analysis was performed to determine genes associated with the exosomal long noncoding (lncRNA) plasmacytoma variant translocation 1 (PVT1)/vascular endothelial growth factor A (VEGFA) axis in patients with colon cancer. The biological importance of the exosomal lncRNA PVT1/VEGFA axis was examined in vitro by using HCT116 and LoVo cell lines and in vivo by using a patient-derived xenograft (PDX) mouse model through knockdown (by silencing of PVT1) and overexpression (by adding serum exosomes isolated from patients with distant metastasis (M-exo)).

**Results:**

The *in silico* analysis demonstrated that PVT1 overexpression was associated with poor prognosis and increased expression of metastatic markers such as VEGFA and epidermal growth factor receptor (EGFR). This finding was further validated in a small cohort of patients with colon cancer in whom increased PVT1 expression was correlated with colon cancer incidence, disease recurrence, and distant metastasis. M-exo were enriched with PVT1 and VEGFA, and both migratory and invasive abilities of colon cancer cell lines increased when they were cocultured with M-exo. The metastasis-promoting effect was accompanied by increased expression of Twist1, vimentin, and MMP2. M-exo promoted metastasis in PDX mice. *In vitro* silencing of PVT1 reduced colon tumorigenic properties including migratory, invasive, colony forming, and tumorsphere generation abilities. Further analysis revealed that PVT1, VEGFA, and EGFR interact with and are regulated by miR-152-3p. Increased miR-152-3p expression reduced tumorigenesis, where increased tumorigenesis was observed when miR-152-3p expression was downregulated.

**Conclusion:**

Exosomal PVT1 promotes colon cancer metastasis through its association with EGFR and VEGFA expression. miR-152-3p targets both PVT1 and VEGFA, and this regulatory pathway can be explored for drug development and as a prognostic biomarker.

## 1. Introduction

Colon cancer is one of the most prevalent malignancies globally and has consistently ranked in the top three leading causes of cancer-associated death [1]. Treating distant metastasis is one of the most challenging tasks in clinical practice. The liver (approximately 20%–30%) and lungs are the most frequently observed metastatic sites in patients with colon cancer [2]. Despite advancements in the development of chemotherapeutic and targeted therapeutic agents over the past decade, the treatment of metastatic colon cancer remains highly difficult. Therefore, obtaining a better understanding of the molecular and signaling mechanisms involved in the metastatic progression of colon cancer, especially epithelial-to-mesenchymal transition (EMT), can help in designing and developing effective therapeutic agents.

Exosomes or extracellular vesicles are nanosized (30–150 nm) cargos produced by cells for intracellular communication [3]. Because of the presence of proteins, lipids, and nucleic acids in exosomes [3] and the functional attributes of exosomes in tumorigenesis, they have received increasing attention. Accumulating evidence suggests that cancer cells secrete exosomes enriched with various signaling molecules to promote tumor initiation, angiogenesis, distant metastasis, and the development of drug resistance [3]. Various studies have confirmed that noncoding RNAs (ncRNAs) are involved in the development of various cancers through regulation of cell differentiation, proliferation, apoptosis, necrosis, and autophagy [4]. In the past few decades, six ncRNAs have been determined to possess no significant protein-coding potential. Two main members of the ncRNA family, long ncRNA (lncRNA) and microRNA (miRNA), were identified to play key roles in regulating the physiology and pathophysiology of cancer [5]. The structure of lncRNA is similar to that of mRNA except that it contains >200 nucleotides; additionally, some lncRNAs have poly(A) tails. As a miRNA sponge, lncRNAs can negatively regulate miRNA expression through sequence-specific binding. By contrast, miRNAs can also negatively regulate the expression of lncRNAs [6]. A recent study revealed that colon cancer cells secrete exosomes containing an ncRNA, namely, miR-193a, which interacts with major vault protein to promote cancer progression [7]. Moreover, these exosomes can be detected in serum, thus making them ideal prognostic markers. Furthermore, although ncRNA molecules were once considered to be evolutionary remnants, they are now understood to play vital roles in every aspect of cellular activity by performing crucial regulatory functions and providing specificity in gene expression. In this study, we examined plasmacytoma variant translocation 1 (PVT1), an lncRNA locus, which has been identified as a candidate oncogene in several types of tumor, including colon cancer tumors [8]. The abnormal proliferation of tumor cells is a crucial feature that distinguishes tumor tissue from normal tissues. Abnormal proliferation of cells is characterized by cell cycle changes, apoptosis inhibition, and disrupted energy metabolism. PVT1, as a potential oncogene, can promote tumor proliferation [9]. PVT1 is a crucial oncogenic lncRNA that is highly expressed in cancer cells. Multiple miRNA response elements have been found on PVT1, and PVT1 can bind to specific miRNAs, thereby silencing them and upregulating the expression of certain proteins, ultimately affecting tumor cell proliferation, invasion, and drug resistance [10, 11]. At present, the carcinogenic effect of PVT1 has been confirmed in various cancers such as gallbladder, non-small-cell lung, and colon cancers [12–14]. Therefore, the invasive and metastatic abilities of cancer cells are assessed for cancer staging and prognosis. A study reported that PVT1 is involved in the EMT and distant metastasis of cancer cells as follows [15]. First, the adhesion between tumor cells and surrounding tissues changes and affects the cells and leads to the primary detachment of cells. Then, the degradation of the extracellular matrix occurs. Furthermore, modification of the cytoskeleton enhances the motility of cancer cells, thereby promoting angiogenesis in tumor tissues. In addition, PVT1 acts as a molecular sponge for miRNAs, which can be trimmed and processed into multiple miRNAs (miR-1204, 1205, 1206, 1207-3p, 1207-5p, and 1208) [10]. These miRNAs can regulate tumor development. For example, overexpression of miR-1207-5p reduces the expression of signal transducers and activators of transcription, thereby activating cyclin-dependent kinase inhibitors (CDKN)1A and CDKN1B that regulate the cell cycle and promote tumor cell proliferation [16].

An adequate understanding of the role of EMT and regulatory mechanisms through which EMT promotes distant metastasis in colon cancer remains elusive. EMT has been confirmed to result in distant metastasis and to cause cancer stemness [17, 18]. Therefore, characterizing key molecular players involved in EMT can provide insights into this process and its mechanisms. Notably, numerous EMT markers have been proposed, all of which converge toward angiogenesis, with vascular endothelial growth factor- (VEGF-) associated signaling playing a crucial role in enabling cancer cells to migrate from their primary niche to a secondary site [19]. In addition, EMT has been associated with the emergence of cancer stemness, the initiation of tumors, and the generation of drug-resistant clones [19–21]. Hence, identifying mechanisms or agents that inhibit the members of the VEGF-associated pathway may represent a means to reduce the incidence of distant metastasis. By using the sequencing technology, researchers have classified and sequenced more than 400 tumors based on microarray data obtained from a public data set [22]. These findings can help us analyze the clinical characteristics of colorectal cancer (such as the expression or inhibition of key genes and their effect on overall survival) and identify crucial prognostic markers.

In the present study, we first collected tissues from both healthy individuals and patients with distant metastatic colon cancer and determined that PVT1 expression was significantly increased in patients with metastatic colon cancer. In addition, by analyzing public databases, we determined that exosomes obtained from patients with metastatic colon cancer and patients with disease recurrence contained increased PVT1. Furthermore, we examined the potential of PVT1 to promote cancer development. We observed that PVT1 loss of function resulted in the suppression of colon tumorigenic and metastatic potential, evident in the suppression of colony formation, cell invasion, sphere formation, and related protein expression *in vitro*. First, we demonstrated that exosomes obtained from patients with metastasis significantly enhanced the migratory and invasive abilities of human colon cancer cell lines HCT116 and LoVo in association with the increased expression of vascular endothelial growth factor A (VEGFA), vimentin, and MMP2. Notably, exosomes isolated from patients with metastatic colon cancer promoted metastasis in patient-derived *in vivo* xenograft mice (with nonmetastatic tumors). In vitro results indicated that PVT1-silenced HCT116 and LoVo cells exhibited significantly decreased tumorigenic and oncogenic properties, including decreased colony- and tumorsphere-forming abilities, as well as migratory and invasive potential. Finally, we explored the possible regulatory mechanism of PVT1 and observed that miR-152-3p, a tumor suppressor, contains binding sites at the 3′-UTR of PVT1 and VEGFA. We observed that the regulatory mechanism of the miR-152-3p/PVT1/VEGFA axis causes metastasis in colon cancer through the overexpression and inhibition of miR-152-3p in colon cells. Therefore, targeting this signaling axis can help in developing effective therapeutic agents against metastatic colon cancer.

## 2. Materials and Methods

### 2.1. Clinical Sample Collection and Preparation

Written informed consent for the collection of clinical samples was obtained from all participants, and this study was approved by the Medical Ethics Committee of Taipei Medical University-Joint Institutional Review Board (N202104054; Taipei, Taiwan). Serum samples were collected from healthy individuals (control) and patients with treatment-naive primary (P) or metastatic (M) colon cancer based on histological examinations (Table 1). Fresh tissues (tumor, adjacent nontumor, and colon metastasis) obtained from patients with colon cancer were processed within 20 min after resection. The samples were then analyzed and confirmed by two independent pathologists. Venous blood samples from patients were collected, and cell-free serum was isolated using a previously established protocol, which involves initial centrifugation at 1600 × *g* for 10 min, followed by repeat centrifugation at 16000 × *g* for 10 min at 4°C. Samples were either used for exosome isolation immediately or were stored at −80°C.

### 2.2. Cell Culture

The human colon cancer cell lines HCT116 (characteristics: derived from the primary colon ascendens tumor, TGF*β*1+/TGF*β*2+, suitable transfection host, and tumorigenic in nude/immunodeficient mice) and LoVo (characteristics: derived from metastatic colon cancer, Dukes' type C, grade IV, colorectal adenocarcinoma, MYC+/KRAS+/HRAS+/NRAS+, suitable transfection host, and tumorigenic in immunodeficient mice) were obtained from the American Type Culture Collection (Manassas, VA, USA). The cells were recently authenticated through short tandem repeat profiling. Both the cell lines were maintained and passaged in RPMI-1640 medium (Gibco, Carlsbad, CA, USA) supplemented with 10% fetal bovine serum (Gibco) in a 5% CO_2_ humidified incubator at 37°C until 90% confluence. Supernatants were collected and centrifuged at 1600 × *g* for 10 min, followed by repeat centrifugation at 16000 × *g* for 10 min at 4°C, and then stored at −80°C until exosome extraction.

### 2.3. Isolation of Exosomes

The serum and culture medium samples were first filtered through a 0.45 *μ*m pore polyvinylidene fluoride filter (Millipore, Darmstadt, Germany). ExoQuick solution (System Biosciences, Palo Alto, CA) was added to the serum samples and incubated at room temperature for 30 min, and ExoQuick-TC solution was added to the culture medium samples and incubated at 4°C for 12 h. Exosomes were sedimented and collected through centrifugation (1500 × *g*, 30 min). The resultant exosome pellets were resuspended in 25 *μ*L of phosphate-buffered saline (PBS).

### 2.4. Transmission Electron Microscopy

Exosomes were diluted to a final concentration of 0.5 mg/mL by using PBS. Exosomes were spotted onto a glow-discharged copper grid and then dried. The samples were stained with a drop of 1% phosphotungstic acid for 5 min and then dried. Morphological analysis of exosomes was performed using a transmission electron microscope (FEI Tecnai, Hillsboro, Oregon) at 200 keV.

### 2.5. RNA Preparation

Total RNA was isolated from the samples (tumor chunks and cell lines) by using TRIzol reagent (Life Technologies, Carlsbad, CA, USA) and quantified using NanoDrop (Thermo Fisher Scientific, Waltham, MA, USA). RNA from exosomes was extracted using an miRNeasy micro kit (QIAGEN, Hilden, Germany). In brief, the exosome suspension (20 *μ*L) was mixed with QIAzol lysis buffer (700 *μ*L) and processed according to the manufacturer's protocol. The RNA samples were subsequently eluted with 25 *μ*L of RNase-free water (repeated twice with the same 25 *μ*L of RNase-free water to concentrate the samples). The RNA concentration in the samples was again determined using NanoDrop.

### 2.6. Real-Time Quantitative Reverse-Transcription Polymerase Chain Reaction

Reverse transcription of miRNAs and real-time quantitative reverse-transcription polymerase chain reaction- (qRT-PCR-) based quantification of miRNA levels were performed using the miDETECT A Track miRNA qRT-PCR Starter Kit (RiboBio, Tokyo, Japan). For PVT1 gene expression analysis, the first strand of cDNA was generated using a PrimeScript first-strand cDNA synthesis kit (TaKaRa, Tokyo, Japan). qRT-PCR was performed using the SYBR Premix Ex Taq II kit (Takara, Tokyo, Japan) on a CFX96 real-time PCR detection system (Bio-Rad, Hercules, CA). The qPCR primers used in this study are listed in Supplementary Table 1.

### 2.7. Migration and Invasion Assays

Colon cancer cells were seeded and cultured in six-well plates for 24 h. The cells were incubated with mitomycin (10 *μ*g/mL) for 1 h. A linear scratch was created through the cell monolayer by using a 200 *μ*L pipette tip. Cellular debris was removed, and the cells were allowed to migrate for 24–48 h. Gap healing was determined from micrographs taken before and after the wound creation under a microscope (Nikon, Japan). Migration distance was measured from images (three random fields) obtained at indicated time points. The gap size was subsequently analyzed using ImageJ software. The invasion assay was performed according to a previously established protocol. In brief, 3 × 10^5^ colon cancer cells were seeded onto Matrigel (BD Biosciences, San Jose, CA, USA) in culture plate inserts (pore size: 8 *μ*m, Corning) in a serum-free medium. Three independent and random fields per well were photographed, and the number of cells in each field was counted. An average of three determinations was obtained for each chamber. Each invasion assay was performed a minimum of three times.

### 2.8. Tumor Spheroid Formation Assay

Colon cancer cells were transferred into serum-free low-adhesion culture plates containing Dulbecco's modified Eagle's medium/F-12 with N2 supplement (Invitrogen), 20 ng/mL of EGF, and 20 ng/mL of basic-FGF (stem cell medium; PeproTech, Rocky Hill, NJ, USA) for 2 weeks to allow tumorsphere formation. The spheres were counted under a microscope. The tumor ball formation efficiency was calculated as the ratio of the number of balls to the number of implanted cells.

### 2.9. Cell Transfection

Colon cancer cells (cell lines or clinical samples) were cultured and maintained as described in previous sections. Gene manipulation experiments, namely, PVT1 silencing experiments, were performed using the siRNA technique (Academia Sinica, Taiwan) according to the manufacturer's instructions. Reagents for the miR-152-3p mimic, inhibitor (oligonucleotides), si-PVT1, and their negative controls (si-NC) were purchased from RiboBio (Academia Sinica, Taiwan), and transfection procedures were performed using Lipofectamine 2000 (Invitrogen, Carlsbad, CA, USA) according to the manufacturer's instructions. The transfected cells were maintained in RPMI-1640 medium (Gibco, Carlsbad, CA, USA) supplemented with 10% fetal bovine serum (Gibco) in a 5% CO_2_-humidified incubator at 37°C. The cells were washed three times in PBS (pH 7.4) before transfection to remove the culture medium and serum.

### 2.10. Western Blot

Total cellular protein lysates from colon cancer cells were extracted using RIPA buffer (Millipore, Darmstadt, Germany), and the lysate concentration was determined using a BCA protein assay kit (Pierce, Rockford, IL, USA). Thirty micrograms of protein were dissolved in SDS-PAGE and transferred onto a PVDF membrane (Millipore, Darmstadt, Germany). After blocking at 37°C for 1 h, the membranes were immunoblotted with different antibodies at 4°C overnight. All antibodies were purchased from Cell Signaling Technology (Danvers, MA) unless otherwise specified. Epidermal growth factor receptor (EGFR) (#2232, 1 : 400), MMP2 (#13667, 1 : 300), vimentin (#5741, 1 : 500), Twist1 (#46702, 1 : 400), GAPDH (#D16H11, 1 : 2000), VEGFA (ab52917, 1 : 500, Abcam, USA), and exosomal markers CD9 (5G6, 1 : 200) and MCT1 (P14612, 1 : 500, Invitrogen, USA) were purchased from Novus Biologicals (Centennial, CO, USA).

### 2.11. *In vivo* Mouse Studies

Six 8-week-old female nonobese diabetic (NOD)/severe combined immunodeficient (SCID) mice obtained from BioLASCO Taiwan Co. Ltd. (Taipei, Taiwan) were bred under standard experimental pathogen-free conditions. All animal experiments were performed according to protocols approved by the experimental animal welfare committee of our institute (approval number: LAC-2020-0535). For the patient-derived xenograft experiment, pieces of tumor mass (approximately 0.05 cm^3^ each, obtained from a patient diagnosed as having colon cancer, and nonmetastatic) were subcutaneously implanted into NOD/SCID mice. Tumor growth (until the tumor became palpable) was allowed in tumor-bearing mice, and exosomes isolated from patients with lung metastasis (M-exo) or primary colon cancer (nonmetastatic, P-exo) were systematically injected through the lateral tail vein (20 *μ*g/mouse, 3 times/week, for 4 weeks); each group contained 10 mice. Tumor growth was monitored and measured using a standard caliper weekly. The tumor volume was determined using the following formula: tumor volume = (width)^2^ × length/2. Animals were humanely sacrificed following experiments, and their tumor and tissue samples were collected for further analyses.

### 2.12. Statistical Analysis

SPSS (version 13.0; SPSS Inc., Chicago, IL) was used to perform all statistical analyses. Each experiment was performed three times. All data in figures are expressed as the mean ± standard deviation (SD). Comparisons between two groups were performed using the *t*-test. All statistical tests were two-sided, and *p* < 0.05 was considered significant. Continuous data were analyzed using the paired samples *t*-test or the Wilcoxon rank test. Categorical data were analyzed using the chi-square test or Fisher's exact test. Survival analysis was estimated using the Kaplan–Meier method along with the log-rank test to calculate differences between the curves.

## 3. Results

### 3.1. PVT1 Is Highly Expressed in Colon Cancer Cells and Affects the Survival Rate

**Figure 1:**
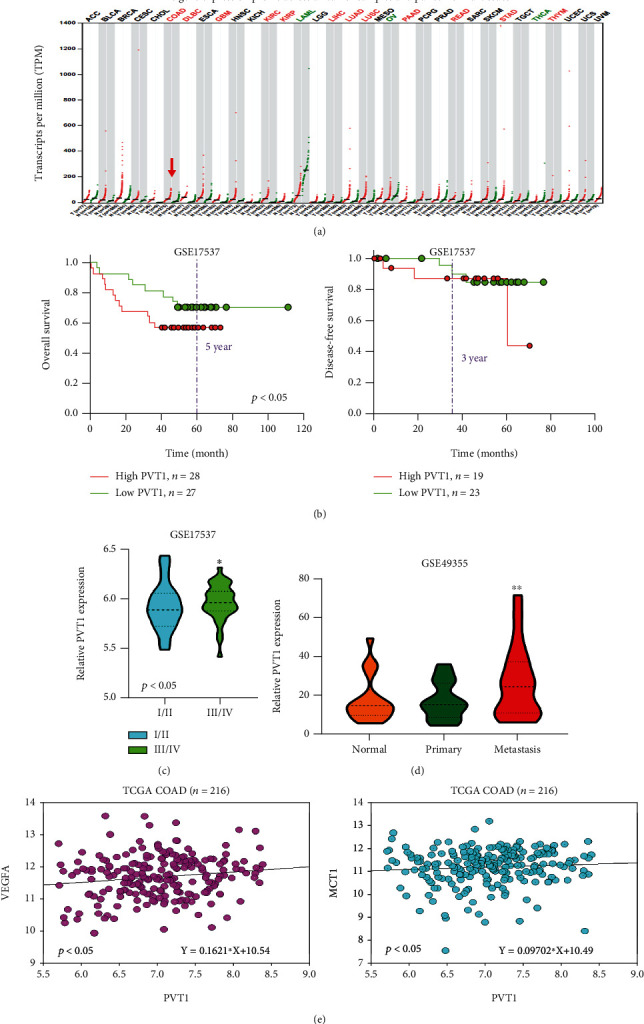
Expression analysis of PVT1 in clinical samples of patients with colon cancer. (a) Expression of PVT1 in the pan-cancer analysis from the database of TCGA. The red arrowhead indicates colon adenocarcinoma. (b) The Kaplan–Meier survival curve and disease-free survival durations constructed from the GSE17537 database. Patients with higher PVT1 expression showed a significantly lower disease-specific survival rate. (c) PVT1 expression in different stages of colorectal cancer. The expression of PVT1 in advanced colorectal cancer is significantly upregulated (*p* < 0.05). (d) The difference in PVT1 expression between primary and metastatic tumors from the GSE49355 dataset. (e) The correlation analysis between PVT1 and VEGFA, PVT1, and the exosome biomarker MCT-1 from the database of the TCGA. *r*: Pearson's correlation index; ^∗^*p* < 0.05; ^∗∗^*p* < 0.01.

Recent studies have revealed PVT1 to be an oncogenic marker for multiple cancer types. Our analysis of a pan-cancer cohort from the Cancer Genome Atlas (TCGA) public cancer database showed that patients with colorectal cancer exhibited a higher PVT1 expression level (Figure 1(a)). We found that patients with colon cancer (GSE17537) with higher PVT1 expression had significantly shorter overall and disease-free survival durations (Figure 1(b)). Further analysis indicated that the expression of PVT1 in advanced colorectal cancer was more significant (Figure 1(c)). Because advanced colorectal cancer can be defined as colorectal cancer that metastasizes when it appears or recurs, we analyzed the difference in PVT1 expression between primary and metastatic tumors from the GSE49355 dataset. The results revealed that the expression level of PVT1 was higher in metastatic tumors (Figure 1(d)). Finally, we performed a correlation analysis between PVT1 and VEGFA data from the database of TCGA. The results indicated that PVT1 was positively correlated with VEGFA and the exosome biomarker MCT-1 (Figure 1(e)). These results suggest a relationship between the exosomal lncRNA PVT1/VEGFA axis and metastatic colorectal cancer. We investigated the effect of confounding factors such as age, sex, tumor size, and tumor stage on survival by using the GSE17537 dataset. The results revealed that age (*X*^2^ = 2.229, *p* = 0.135) and primary tumor stage (*X*^2^ = 0.900, *p* = 0.343) were not significantly associated with PVT1 expression; however, female patients demonstrated higher PVT1 expression than male patients (*X*^2^ = 5.238, *p* = 0.022; Supplementary Table 2).

### 3.2. Serum Exosomes Isolated from Patients with Metastatic Colon Cancer Promoted Metastatic Potential in Nonmetastatic Colon Cancer Cells

**Figure 2:**
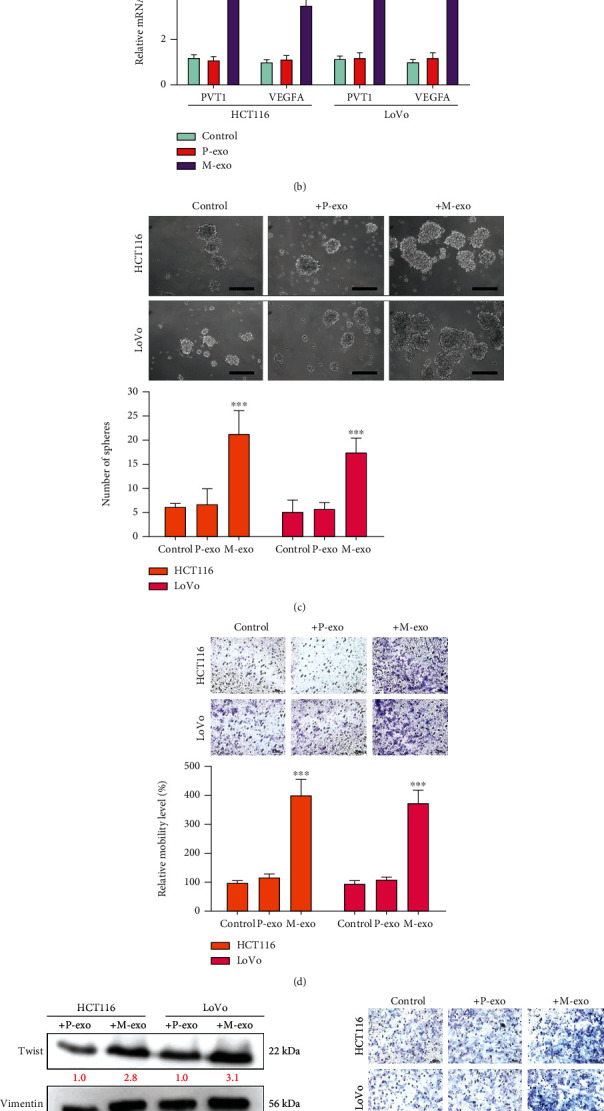
Comparative analysis of exosomes from the serum of patients with primary and metastatic colon cancer. (a) Serum exosomes were isolated from patients with primary tumor (P-exo) and distant metastasis (M-exo). Representative electromagnetic images of exosomes are shown. Scale bar: 200 nm. Western blots demonstrated increased CD9 and MCT1 (exosome markers) and cyclin D1 (tumor-specific marker) in the serum of patients with metastatic colon cancer (M, M-exo) compared with that of patients with primary tumor (P, P-exo). Serum exosomes of normal healthy people were included as controls (N, normal). (b) Comparative qPCR analysis showed that the levels of PVT1 and VEGFA were significantly higher in the M-exo than in the P-exo. (c) The sphere-forming assay showed that the addition of M-exo led to formation of an increased number of tumorspheres in both cell lines compared with controls and the group with P-exo. (d) Exosomes and colon cancer cell line coculture experiment. HCT116 and LoVo cells cocultured with M-exo demonstrated enhanced migratory and invasive abilities. (e) Western blot analysis indicated an elevation in metastatic markers, namely, Twist1, vimentin, and MMP2, and the stemness marker Sox2 in HCT116 and LoVo cells cocultured with M-exo compared with their counterparts cultured with P-exo. Coculture with exosomes derived from the serum of healthy individuals served as control. ^∗^*p* < 0.05; ^∗∗^*p* < 0.01; ^∗∗∗^*p* < 0.001. Scale bar: 100 *μ*m.

Table 1 shows the relevant clinicopathological information, including age, sex, and clinical stage, of patients with treatment-I primary or metastatic colon cancer. The results showed that distant metastasis (*X*^2^ = 4.552, *p* = 0.033) and primary stage (*X*^2^ = 4.000, *p* = 0.045) were significantly associated with PVT1 expression. Because we noted an increased PVT1 level in the tissue or serum samples of patients with colon cancer, we analyzed serum exosomes isolated from these patients. In the representative micrographs, P-exo and M-exo denote exosomes isolated from primary (nonmetastatic) and metastatic samples, respectively. Serum exosomes isolated from normal healthy people were included as the control (normal (N)). The average size of exosomes ranged from 100 to 200 nm. In addition, we detected the expression levels of three tumor-specific biomarkers, namely, CD9, MCT1, and cyclin D1, in exosomes. Western blot results indicated increased expression of CD9 and MCT1 (exosome markers) in the serum samples of patients with metastatic colon cancer compared with those of patients with primary tumor (Figure 2(a)). We cultured human colon cancer cells, namely, HCT116 and LoVo, with P-exo and M-exo under serum-deprived conditions. Subsequently, the findings of qPCR showed that M-exo contained significantly higher levels of PVT1 and VEGFA compared with P-exo (Figure 2(b)). In addition, we found that both cell lines exhibited increased ability to form tumorspheres under the presence of M-exo compared with P-exo (Figure 2(c)). Furthermore, the migratory and invasive abilities of HCT116 and LoVo were significantly increased in the presence of M-exo but not P-exo (Figure 2(d)). The Western blots of both HCT116 and LoVo cells cultured with M-exo demonstrated an increase in EMT markers, namely, Twist1, vimentin, and MMP2, as well as the stemness marker Sox2 compared with HCT116 and LoVo cells cultured with P-exo (Figure 2(e)). In addition, we examined the effects of adding two serum exosomes on the growth of different types of cancer cells, namely, U87 (a human primary glioblastoma cell line), SAS (human squamous cell carcinoma of the tongue), MDA-MB-231 (a triple-negative breast cancer cell line), and HCT116 (a colon cancer cell line), as controls. The results indicated that the cells cultured with M-exo demonstrated increased cell viability (Supplementary Figure S1).

### 3.3. M-exo Promoted Distant Metastasis in a Patient-Derived Xenograft Mouse Model

**Figure 3:**
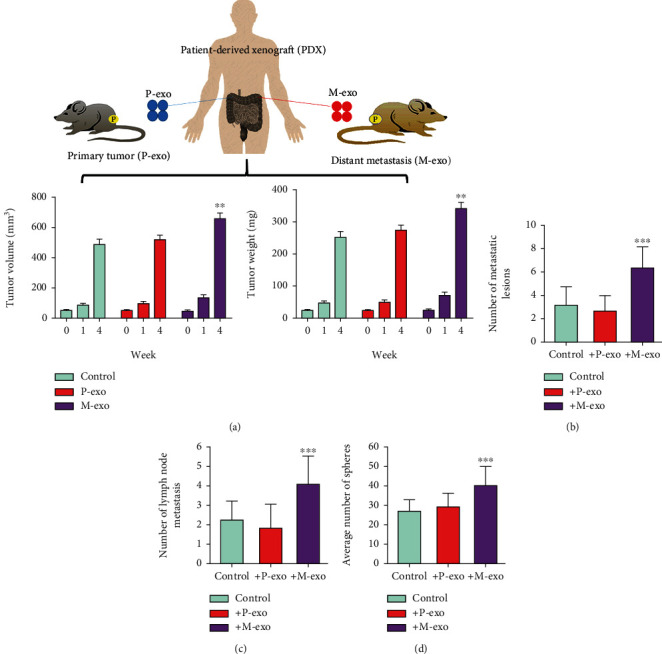
M-exo promoted tumor growth and distant metastasis in the PDX mouse model. (a) PDX mice (*n* = 10) were divided into the following groups: control, M-exo, and P-exo. The M-exo group showed a higher tumor volume and weight (week 4) than did control and P-exo groups. Distant (b) lung and (c) lymph node metastasis analysis. Lesions in the lungs from all the three groups were examined and counted. The M-exo group exhibited the highest number of lung lesions, followed by P-exo and control groups. (d) Comparative analysis of tumorsphere-forming ability. Tumor samples harvested from the M-exo group showed a higher number of spheres; no significant difference in the number of spheres was observed between the P-exo and control groups. Mice inoculated with cells cocultured with exosomes derived from the serum of healthy individuals served as control. ^∗^*p* < 0.05; ^∗∗^*p* < 0.01; ^∗∗∗^*p* < 0.001.

Patient-derived xenograft (PDX) mouse models were established using NSG mice bearing patient samples from primary (nonmetastatic) colon cancer tumors. Serum exosomes, M-exo, and P-exo were intravenously injected 1 week following tumor implantation. Injections were administered three times a week for 4 weeks. PDX mice injected with M-exo exhibited significantly higher tumor and tissue growth (Figure 3(a)) and more distant lesions in the lungs compared with other groups (Figure 3(b)); a similar observation was made for lymph node metastasis (Figure 3(c)). In addition, we compared the tumorsphere generation ability among the groups and found that tumor cells harvested from M-exo mice exhibited an enhanced ability to form tumorspheres in terms of both number and size (Figure 3(d)); the number of spheres formed in mice injected with P-exo did not significantly differ from that formed in control mice.

### 3.4. Inhibition of PVT1 Suppresses the Tumorigenic Properties of Colon Cancer Cells

**Figure 4:**
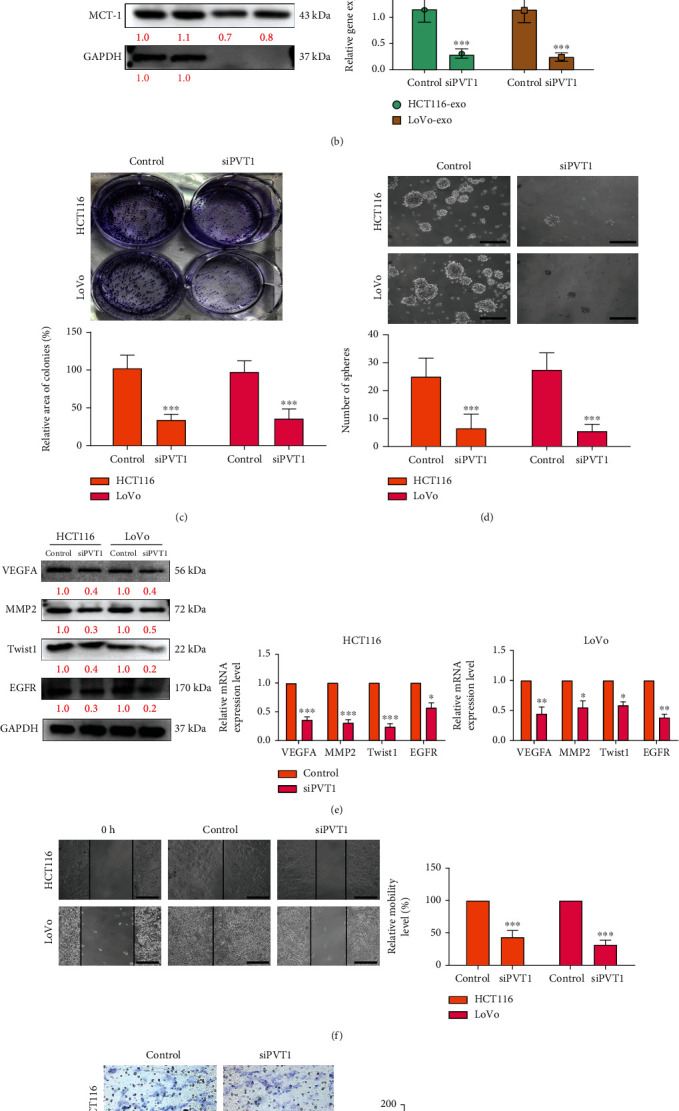
PVT1 silencing suppressed colon tumorigenic and metastatic potential. (a) The siRNA knockdown effect of PVT1 on two colon cancer cell lines. (b) The basal levels of PVT1 and VEGF (Western blot and gene expression) in cell lysates and exosomes. (c) Colony formation assay revealed that si-PVT1-transfected HCT116 and LoVo cells formed a significantly lower number of colonies compared with control parental cells. (d) Comparative tumorsphere-forming assay. HCT116 and LoVo cells transfected with si-PVT1 were significantly less potent in forming tumorspheres compared with their parental cells. (e) Comparison of expression between parental colon cancer cells and PVT1-silenced cells. Right panels: qPCR analysis demonstrated markedly decreased expression of metastatic markers, namely, VEGFA, Twist1, and MMP2, and the oncogenic marker EGFR in si-PVT1 colon cells. Left panels: Western blots of parental versus PVT1-silenced HCT116 and LoVo cells. Prominent reduction in the expression of VEGFA, Twist1, MMP2, and EGFR was observed after PVT1 silencing in both cell lines. Effect of PVT1 expression on cell (f) migration and (g) invasion of HCT116 and LoVo cells detected using Transwell assays. NC: negative control (scramble PVT1 oligonucleotides). ^∗^*p* < 0.05; ^∗∗^*p* < 0.01; ^∗∗∗^*p* < 0.001. Scale bar: 100 *μ*m.

We examined the functional roles of PVT1 in colon cancer by silencing PVT1 through the siRNA technique.  Figure 4(a) shows the siRNA knockdown effect of PVT1 on two colon cancer cell lines.  Figure 4(b) presents the basal levels of PVT1 and VEGF (Western blot and gene expression) in cell lysates and exosomes. Supplementary Figure S2 shows the basal levels of the PVT1 and VEGF family in different cancer cell lines, namely, U87, FaDu (hypopharyngeal tumor), MDA-MB-231, and pHCT116 (a colon cancer cell line), that were included as controls. The data were obtained from the Gene Expression Omnibus (GEO) database (GSE36133). We observed that PVT1 promoted angiogenesis by regulating the VEGF signaling pathway in different cancer cells. PVT1-silenced HCT116 and LoVo cells exhibited significantly decreased colony-forming ability (Figure 4(c)). For example, PVT1-silenced LoVo cells formed approximately 70% fewer colonies than their nonsilenced counterparts (Figure 4(c)). HCT116 and LoVo cells transfected with si-PVT1 were significantly less potent in forming tumorspheres compared with their parental cells (Figure 4(d)). Our bioinformatics research was supported by the fact that PVT1-silenced HCT116 and LoVo cells exhibited significantly lower mRNA (right panels) and protein expression levels of metastatic markers, namely, VEGFA, MMP2, and Twist1, and the oncogenic marker EGFR (left panels; Figure 4(e)). These results indicate markedly suppressed migratory (Figure 4(f)) and invasive abilities (Figure 4(g)). The downregulation of PVT1 suppressed the migration of HCT116 cells by approximately 50% (*p* < 0.01) and that of LoVo cells by approximately 55% (*p* < 0.01); the invasive ability was reduced by at least 50% (*p* < 0.01) in both HCT116 and LoVo cells after PVT1 silencing.

### 3.5. Tumor Suppressor miR-152-3p Inhibits the Expression of PVT1 and the Metastatic Potential of Colon Cancer

**Figure 5:**
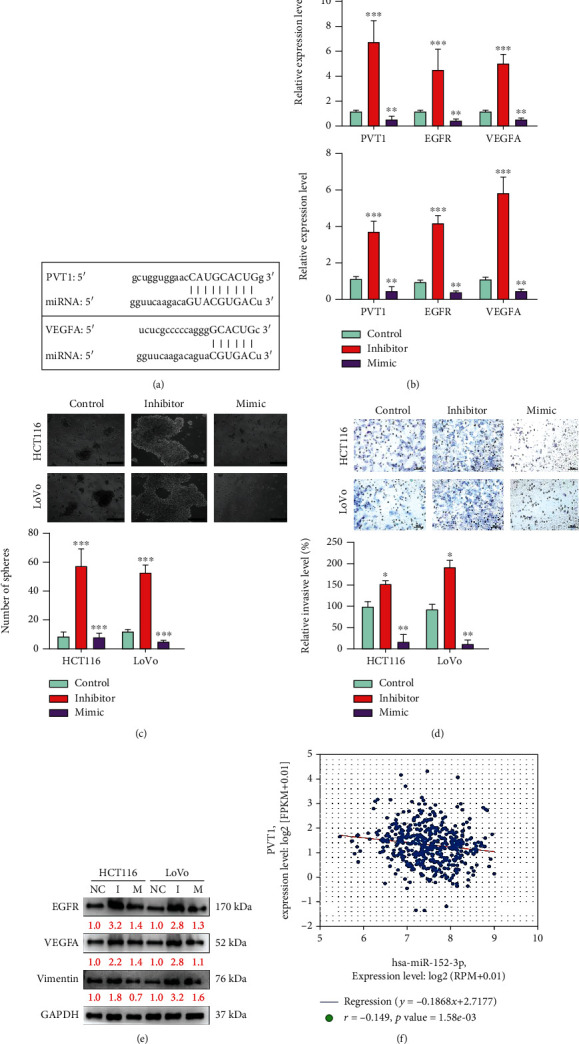
Target validation for miR-152-3p and its role in suppressing metastasis. (a) Target binding sequences of miR-152-3p in the 3′-UTR of PVT1 and VEGFA. These binding sites were predicted using both miRmap and MiRanda software. (b) qPCR analysis of PVT1, EGFR, and VEGFA levels in response to the sequential miR-152-3p mimic and inhibitor transfections (the control group did not add any reagents). A significant decrease in the mRNA levels of PVT1, EGFR, and VEGFA was observed after miR16-5p mimic transfection and subsequent restoration with the addition of the miR-152-3p inhibitor. Both HCT116 and LoVo cells showed a similar trend. (c) Tumorsphere formation assay. The tumorsphere-forming ability was considerably inhibited by the transfection of miR-152-3p in both HCT116 and LoVo cells; partial restoration of the tumorsphere-forming ability was noted when the miR-152-3p inhibitor was added. (d) Invasion assay revealed that an increase in miR-152-3p significantly reduced the invasive ability in both HCT116 and LoVo cells; however, the invasive ability was restored by the addition of the miR-152-3p inhibitor. (e) Western blot analysis. The addition of miR-152-3p mimics suppressed the expression of EGFR, vimentin, and VEGFA in both HCT116 and LoVo cells, and the inhibitor restored their expression. (f) A negative correlation was noted between miR-152-3p and PVT1 levels in colon cancer clinical samples from databases of TCGA [12] (*n* = 450). Control/NC: scramble hsa-miR-152-3p oligonucleotides. ^∗^*p* < 0.05; ^∗∗^*p* < 0.01; ^∗∗∗^*p* < 0.001. Scale bar: 100 *μ*m.

By examining different online platforms, namely, Ensembl (https://www.ensembl.org/), LNCipedia (https://lncipedia.org/), miRDB (http://mirdb.org/), and the ENCORI pan-cancer analysis platform (http://starbase.sysu.edu.cn/), we determined that PVT1 and VEGFA are targeted by miR-152-3p, which was identified as a potential inhibitor of PVT1 and VEGFA [23]. The binding sequences are shown in Figure 5(a). The expression of miR-152-3p was higher than that of PVT1 in the early stage, and the expression of PVT1 was higher than that of miR-152-3p in the late stage (Supplementary Figure S3). On the basis of this finding, we examined the effects of the miR-152-3p mimic and inhibitor oligonucleotides on colon tumorigenesis. An increased level of miR-152-3p in both HCT116 and LoVo cells led to significantly lower expression of PVT1, VEGFA, and EGFR (Figure 5(b)). Moreover, the mimic-induced overexpression of miR-152-3p significantly reduced tumorsphere formation in both cell lines (Figure 5(c)). However, the subsequent addition of the miR-152-3p inhibitor significantly restored tumorsphere formation ability (Figure 5(c)). A similar observation was made for invasive ability. The invasive ability of HCT116 and LoVo cells was significantly reduced when the cells were transfected with the miR-152-3p mimic and oligonucleotides (Figure 5(d)). The invasive ability was restored by the subsequent addition of the miR-152-3p inhibitor (Figure 5(d)). These findings are supported by the results of Western blot analysis that revealed decreased levels of metastasis-associated markers, namely, VEGFA and vimentin, and the oncogenic marker EGFR in mimic-transfected cells (Figure 5(e), lane M); these levels were restored after the addition of the miR-152-3p inhibitor (Figure 5(e), lane I). Furthermore, the analysis of 450 patients with colon cancer from the database of TCGA revealed a negative correlation between the expression of PVT1 and that of miR-152-3p (coefficient *r* value = −0.149 and *p* = 1.58*E* − 03; Figure 5(f)).

## 4. Discussion

Exosomes are small vesicles secreted by cells and are a type of membrane-bound extracellular vesicle; they can be found in various body fluids and participate in communication between cells [24]. Recent studies have indicated that proteins and related RNAs present in exosomes can be used as biological indicators for cancer diagnosis and prognosis assessment. PVT1 is an oncogenic lncRNA and is associated with many cancer types including colorectal and gastric cancers [12–14]. PVT1 contributes to many aspects of cancer biology through a complex signal network, with a role in tumor growth, metastasis, and response to chemotherapy and radiotherapy [9]. This complex signal network involves interactions with DNA, RNA, and proteins. In the present study, we identified PVT1 as a prognostic biomarker in patients with colon cancer. Notably, patients with a higher PVT1 level exhibited a shorter survival duration than did patients with a lower PVT1 level. In addition, a higher PVT1 level was noted in the serum of patients with recurrent disease. Notably, serum exosomes isolated from patients with metastasis revealed an increased PVT1 level compared with those isolated from patients without metastasis. Moreover, cell line experiments exhibited increased PVT1 levels in HT29 colon spheres compared with their parental counterparts along with increased VEGFA and EGFR levels, the two major metastatic or oncogenic markers in colon cancer. These observations suggest that PVT1 in serum exosomes play a crucial role in promoting metastasis in colon cancer.

Our comparative experiments performed using M-exo and P-exo revealed that M-exo not only contained a significantly higher PVT1 level than did P-exo but also enhanced the migratory and invasive abilities of the human colon cancer cell lines HCT116 and LoVo. This finding indicates that exosomes enriched with PVT1 are one of the venues for colon cancer cells with metastatic ability to transform neighboring cancer cells. Moreover, this phenomenon was observed in non-small-cell lung cancer wherein the lncRNA MALAT1 was found to be protected by exosomes and involved in the promotion of distant metastasis [25]. Concordantly, we noted that serum exosomes isolated from patients with metastatic colon cancer contained a higher PVT1 level than did those isolated from patients with primary nonmetastatic colon cancer. Therefore, PVT1-enriched serum exosomes can be used as a potential new prognostic biomarker for metastatic colon cancer.

Another phenomenon observed in our study was the association between increased PVT1 expression and colon tumorsphere generation. In addition, the findings of in silico analysis showed that the PVT1 level was significantly higher in HT29 tumorspheres. Furthermore, the results of our in vitro experiments revealed that the ability to form tumorspheres was severely compromised when PVT1 expression was inhibited in both HCT116 and LoVo cells. Moreover, M-exo promoted the formation of tumorspheres in our PDX mouse model in vivo. This finding provides an indirect link to the EMT-promoted stemness hypothesis first proposed by Shibue and Weinberg [26]. In the case of colon cancer, the presence of CD26(+) cells in primary tumors could predict distant metastasis at follow-up. Moreover, isolated CD26(+) cells, but not CD26(−) cells, caused distant metastasis when injected into mice [27]. This finding is similar to our in vivo result that PVT1-enriched M-exo promoted distant metastasis in PDX mice inoculated with primary (nonmetastatic) clinical colon cancer cells. These findings indicate that PVT1-enriched exosomes can function as an enhancer of the EMT process and cancer stemness in colon cancer. Nevertheless, the exact mechanistic role of PVT1 in the promotion of stemness warrants further investigation and is currently under exploration in our laboratory.

VEGFA plays a major role in promoting colon metastasis by engaging with Sox2-associated signaling [28]. This finding accords with our observation that the addition of exosomes isolated from metastatic colon cancer promoted the generation of tumorspheres in both HCT116 and LoVo cells in association with increased Sox2 expression. Notably, EGFR and VEGF mutations are often determined to be associated with increased metastatic incidence and poor prognosis in colon cancer [29]. Our observation of PVT1 downregulation leading to decreased expression of VEGFA, EGFR, Twist1, and MMP2 provides insight into the role of PVT1 in promoting metastasis. Our database analysis indicated that the expression levels of PVT1, VEGFA, and EGFR were significantly elevated in HT29 spheres compared with their parental cells [30]. Although the present study did not establish a causal relationship among the expression of these three molecules, a common regulatory molecule, namely, miR-152-3p, was identified to be an inhibitor of both PVT1 and VEGFA. First, we identified the potential binding sites of miR-152-3p to PVT1 and VEGA and observed that increased expression of miR-152-3p significantly reduced the expression of PVT1, VEGFA, and EGFR in both HCT116 and LoVo cells. A previous study indicated that PVT1 was linked to the activation of STAT3/VEGFA signaling and promoted angiogenesis in patients with gastric cancer [31]. miR-152-3p has been identified as a tumor suppressor; for example, miR-152-3p was observed to negatively regulate PIK3CA expression, thus inhibiting the activation of AKT and RPS6 in breast cancer cells [32]. In addition, the downregulation of miR-152, which targets DNMT1 (an oncogene or cancer stemness marker), was observed in breast cancer cells [33]. Notably, a negative correlation between the expression levels of PVT1 and miR-152-3p was identified in TCGA colon cancer database consisting of 450 patient samples [32]. Recent studies have demonstrated that PVT1 regulates the VEGFA/VEGF receptor 1 (VEGFR1)/AKT axis and promotes the tumorigenesis of colorectal cancer; the deletion of PVT1 can reduce tumor volume. Overexpression of VEGFA regulates the AKT signaling cascade by activating VEGFR1 [34]. miR-152-3p has been described as a possible tumor suppressor in numerous studies because its downregulation has been reported in several cancer tissues. Our results indicate that because miR-152-3p may have binding sites for PVT1 and VEGFA, it might regulate the lncRNA PVT1/VEGFA axis. Previous studies have found that PVT1 can promote angiogenesis in tumor tissues. Given the causal relationship between upstream and downstream genes, studies should evaluate whether miR-152-3p can skip PVT1 and directly regulate VEGFA.

Patients with colorectal cancer with high PVT1-214 expression have a shorter survival duration and poor prognosis. The results of in vivo and in vitro studies have indicated that the effects of PVT1-214 exhibit a complex phenotype affecting cell growth, stem-like properties, migration, and invasion [35]. PVT1 can indirectly regulate the expression of four-jointed box 1 (FJX1) by targeting miR-106b-5p. By regulating the miR-106b-5p/FJX1 axis, knockdown of PVT1 can impair cell proliferation, migration, and invasion in colorectal cancer [36]. Many studies have reported that PVT1 overexpression was related to cancer growth and proliferation in vivo and in vitro. Moreover, human tissue and mouse xenotransplantation studies have indicated that the overall survival rate and tumor size of many cancer types are related to PVT1 overexpression [37, 38].

lncRNAs are a major type of ncRNA. In addition, the role of PVT1 in cancer development is closely related to miRNAs. PVT1 can be spliced and processed into various miRNAs such as miR-1204 and miR-1207. Furthermore, PVT1 can act as a “sponge” for miRNAs and inhibit their activity, thereby affecting cancer proliferation, invasion, and angiogenesis [39]. Patients with digestive system cancers have a high risk of morbidity and mortality. The expression of PVT1 is elevated in various digestive system cancers and is associated with poor prognosis [40]. Therefore, PVT1 might become a new biomarker for early screening and could potentially be used as a prognostic biomarker and in future targeted therapies to improve the survival rate of patients [40–42]. Liquid biopsy is a novel method used for cancer diagnosis. Circulating ncRNAs such as miRNAs and lncRNAs are biomarkers that can be easily obtained from blood and can be used for early diagnosis and prediction of prognosis and treatment response. lncRNAs can be passively released through tissues or exosomes [43]. Recently, the therapeutic targeting of oncogenic lncRNAs and lncRNAs related to therapeutic resistance has attracted considerable attention [44]. lncRNAs mediate these functions by interacting with proteins, RNA, and lipids. Unlike other ncRNAs, the functions of lncRNAs cannot be inferred from their sequence or structure [45]. Therefore, experimental evaluations are required to obtain complete and accurate biological annotations. A recent study indicated that lncRNA PVT1 promotes the tumorigenesis of colorectal cancer by stabilizing miR-16-5p and interacting with the VEGFA/VEGFR1/AKT axis [46]. In a mouse xenograft model, the combination of PVT1 loss and miR-16-5p overexpression minimized tumor volume. lncRNA PVT1 acts as a miRNA sponge and negatively regulates miR-16-5p expression. VEGFA is a key regulator of angiogenesis and migration [47]. Angiogenesis is a crucial marker for tumorigenesis, progression, and prognosis [47]. In our study, we discovered the regulatory role of miR-152-3p in the lncRNA PVT1/VEGFA axis, especially exosomes, which are closer to the exploration and application of tumor microenvironment. Our findings regarding the exosomal lncRNA PVT1/VEGFA axis confirms this point. PVT1 in the serum exosomes of patients could affect metastasis and invasion in both in vivo and in vitro models. Exosomes can be used for precise treatment and early diagnosis, thus inspiring novel treatment strategies for colon cancer.

## 5. Conclusion

**Figure 6:**
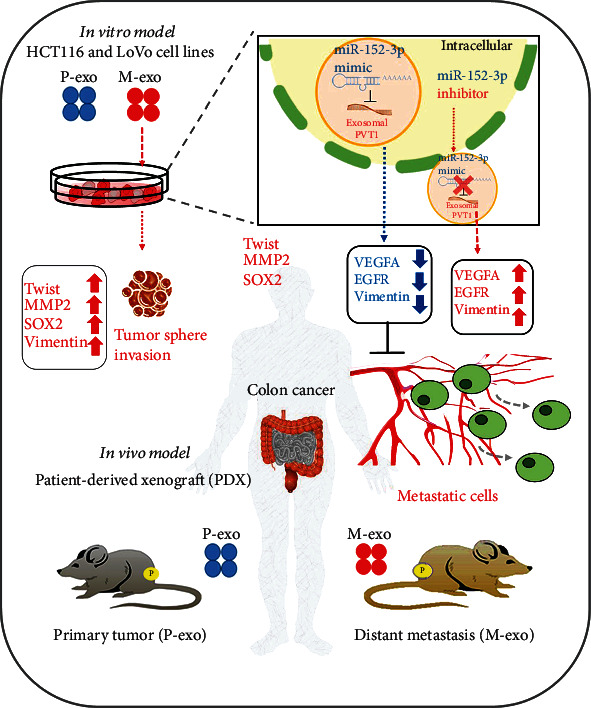
Schematic abstract showing that exosomal PVT1 promotes metastasis in colon cancer through its association with EGFR and VEGFA expression.

Exosomal PVT1 promotes colon cancer metastasis through its association with EGFR and VEGFA expression. miR-152-3p targets both PVT1 and VEGFA, and this regulatory pathway can be explored for drug development and as a prognostic biomarker. A series of experimental designs and their results are shown in the schema abstract in Figure 6. The results of this study indicated the potential role of PVT1/VEGFA-enriched serum exosomes in promoting distant metastasis in colon cancer. Both in vitro and in vivo experiments revealed that increased PVT1 expression was associated with increased metastatic potential and stemness, as reflected by the concomitantly increased expression of VEGFA, EGFR, and Sox2. Moreover, the downregulation of PVT1 significantly suppressed the metastatic ability of colon cancer cells, and PVT1 was observed to be regulated by the tumor suppressor miR-152-3p. Nevertheless, future studies should explore this perspective further and analyze the possibility of designing agents that can increase miR-152-3p expression for treating metastatic colon cancer.

## Figures and Tables

**Table 1:** Clinical table of metastatic patients that increased PVT1 expression and related information.

Clinicopathological variables	No.	PVT1		*X* ^2^	*p* value
		High	Low		
Age, years					
≤55	17	8	9	0.051	0.822
>55	23	10	13		
Gender					
Male	28	15	13	1.380	0.240
Female	12	4	8		
Tumor differentiation					
Well/moderately	19	9	10	3.536	0.060
Poorly	21	16	5		
Lymph node metastasis					
N0	15	5	10	2.667	0.102
N1–N2	25	15	10		
Distant metastasis					
M0	18	8	10	4.552	0.033
M1	22	17	5		
Primary stage					
I + II	13	6	7	4.000	0.045
III + IV	27	21	6		

## Data Availability

The authors confirm that the data supporting the findings of this study are available within the article and/or its each supplementary material files. The manuscript is available as a preprint at the following website: https://www.researchsquare.com/article/rs-116035/v1.

## Abbreviations

CSCs: Cancer stem cells
DTX: Docetaxel
EMT: Epithelial-to-mesenchymal transition
ECM: Extracellular matrix
5-FU: 5-Flurouracil
FBS: Fetal bovine serum
IFC: Immunofluorescence
SRB: Sulforhodamine B
TCA: Trichloroacetic acid
TICs: Tumor-initiating cells.

## Additional Points

*Highlights.* (i) Serum exosomes enriched with lncRNA PVT1/VEGFA in colon cancer. (ii) lncRNA PVT1/VEGFA promotes metastatic potential and stemness. (iii) lncRNA PVT1/VEGF is in association with downregulation of miR-152-3p.

## Supplementary Materials

Supplementary Materials Supplementary Table 1: primer sequences used in this study. Supplementary Table 2: clinical table (GSE17537) of Figure 1(b). Supplementary Figure S1: cell viability of different cancer cell lines after exosome treatment. Supplementary Figure S2: gene expression of the PVT1 and VEGF family in different cancer cell lines. Supplementary Figure S3: relative gene expression of miR-152-3p and PVT1 in primary stages. Supplementary Figure S4: full-size blots of Figure 2(a). Supplementary Figure S5: full-size blots of Figure 2(e). Supplementary Figure S6: full-size blots of Figure 4(b). Supplementary Figure S7: full-size blots of Figure 4(e). Supplementary Figure S8: full-size blots of Figure 5(e).

## References

[1] Jin K., Lan H., Cao F. (2012). Differential response to EGFR- and VEGF-targeted therapies in patient-derived tumor tissue xenograft models of colon carcinoma and related metastases. *International Journal of Oncology*.

[2] Benson A. B., Venook A. P., Cederquist L. (2017). Colon cancer, version 1.2017, NCCN clinical practice guidelines in oncology. *Journal of the National Comprehensive Cancer Network: JNCCN*.

[3] Colombo M., Raposo G., Thery C. (2014). Biogenesis, secretion, and intercellular interactions of exosomes and other extracellular vesicles. *Annual Review of Cell and Developmental Biology*.

[4] Chi Y., Wang D., Wang J., Yu W., Yang J. (2019). Long non-coding RNA in the pathogenesis of cancers. *Cell*.

[5] Zhao M., Zhu N., Hao F. (2019). The regulatory role of non-coding RNAs on programmed cell death four in inflammation and cancer. *Frontiers in Oncology*.

[6] Huang Y. (2018). The novel regulatory role of lncRNA-miRNA-mRNA axis in cardiovascular diseases. *Journal of Cellular and Molecular Medicine*.

[7] Teng Y., Ren Y., Hu X. (2017). MVP-mediated exosomal sorting of miR-193a promotes colon cancer progression. *Nature Communications*.

[8] Tseng Y. Y., Moriarity B. S., Gong W. (2014). _PVT1 dependence in cancer with MYC copy-number increase. *Nature*.

[9] Onagoruwa O. T., Pal G., Ochu C., Ogunwobi O. O. (2020). Oncogenic role of PVT1 and therapeutic implications. *Frontiers in Oncology*.

[10] Luo Z., Cao P. (2019). Long noncoding RNA PVT1 promotes hepatoblastoma cell proliferation through activating STAT3. *Cancer Management and Research*.

[11] Xu Y., Luo X., He W. (2018). Long non-coding RNA PVT1/miR-150/ HIG2 axis regulates the proliferation, invasion and the balance of iron metabolism of hepatocellular carcinoma. *Cellular Physiology and Biochemistry*.

[12] Chen J., Yu Y., Li H. (2019). Long non-coding RNA PVT1 promotes tumor progression by regulating the miR-143/HK2 axis in gallbladder cancer. *Molecular Cancer*.

[13] Li X., Zhang Z., Jiang H. (2018). Circular RNA circPVT1 promotes proliferation and invasion through sponging miR-125b and activating E2F2 signaling in non-small cell lung cancer. *Cellular Physiology and Biochemistry*.

[14] Yu X., Zhao J., He Y. (2018). Long non-coding RNA PVT1 functions as an oncogene in human colon cancer through miR-30d-5p/RUNX2 axis. *Journal of BUON*.

[15] Wu Y., Shao A., Wang L. (2019). The role of lncRNAs in the distant metastasis of breast cancer. *Frontiers in Oncology*.

[16] Yan C., Chen Y., Kong W. (2017). PVT1-derived miR-1207-5p promotes breast cancer cell growth by targeting STAT6. *Cancer Science*.

[17] Dongre A., Weinberg R. A. (2019). New insights into the mechanisms of epithelial-mesenchymal transition and implications for cancer. *Nature Reviews Molecular Cell Biology*.

[18] Zhang Y., Weinberg R. A. (2018). Epithelial-to-mesenchymal transition in cancer: complexity and opportunities. *Frontiers of Medicine*.

[19] Fantozzi A., Gruber D. C., Pisarsky L. (2014). VEGF-mediated angiogenesis links EMT-induced cancer stemness to tumor initiation. *Cancer Research*.

[20] Lu H., Clauser K. R., Tam W. L. (2014). A breast cancer stem cell niche supported by juxtacrine signalling from monocytes and macrophages. *Nature Cell Biology*.

[21] Ye X., Tam W. L., Shibue T. (2015). Distinct EMT programs control normal mammary stem cells and tumour- initiating cells. *Nature*.

[22] Teer J. K. (2014). An improved understanding of cancer genomics through massively parallel sequencing. *Translational Cancer Research*.

[23] Kertesz M., Iovino N., Unnerstall U., Gaul U., Segal E. (2007). The role of site accessibility in microRNA target recognition. *Nature Genetics*.

[24] Raposo G., Stoorvogel W. (2013). Extracellular vesicles: exosomes, microvesicles, and friends. *The Journal of Cell Biology*.

[25] Zhang R., Xia Y., Wang Z. (2017). Serum long non coding RNA MALAT-1 protected by exosomes is up-regulated and promotes cell proliferation and migration in non-small cell lung cancer. *Biochemical and Biophysical Research Communications*.

[26] Shibue T., Weinberg R. A. (2017). EMT, CSCs, and drug resistance: the mechanistic link and clinical implications. *Nature Reviews Clinical Oncology*.

[27] Pang R., Law W. L., Chu A. C. Y. (2010). A subpopulation of CD26^+^ cancer stem cells with metastatic capacity in human colorectal cancer. *Cell Stem Cell*.

[28] Kim M., Jang K., Miller P. (2017). VEGFA links self-renewal and metastasis by inducing Sox2 to repress miR-452, driving Slug. *Oncogene*.

[29] Rigopoulos D. N. (2010). Deregulation of EGFR/VEGF/HIF-1a signaling pathway in colon adenocarcinoma based on tissue microarrays analysis. *Journal of BUON*.

[30] Hwang W.-L., Yang M.-H., Tsai M.-L. (2011). SNAIL regulates interleukin-8 expression, stem cell-like activity, and tumorigenicity of human colorectal carcinoma cells. *Gastroenterology*.

[31] Zhao J., du P., Cui P. (2018). LncRNA PVT1 promotes angiogenesis via activating the STAT3/VEGFA axis in gastric cancer. *Oncogene*.

[32] Li J. H., Liu S., Zhou H. (2014). starBase v2.0: decoding miRNA-ceRNA, miRNA-ncRNA and protein-RNA interaction networks from large-scale CLIP-Seq data. *Nucleic Acids Research*.

[33] Li Z., Li Y., Li Y. (2017). Long non-coding RNA H19 promotes the proliferation and invasion of breast cancer through upregulating DNMT1 expression by sponging miR-152. *Journal of Biochemical and Molecular Toxicology*.

[34] Liu H., Yin Y., Liu T. (2021). Long non-coding RNA PVT1 regulates the migration of hepatocellular carcinoma HepG2 cells via miR-3619-5p/MKL1 axis. *Bosnian Journal of Basic Medical Sciences*.

[35] He F., Song Z., Chen H. (2019). Long noncoding RNA PVT1-214 promotes proliferation and invasion of colorectal cancer by stabilizing Lin28 and interacting with miR-128. *Oncogene*.

[36] Liu F., Wu R., Guan L., Tang X. (2020). Knockdown of PVT1 suppresses colorectal cancer progression by regulating miR-106b-5p/FJX1 axis. *Cancer Management and Research*.

[37] Derderian C., Orunmuyi A. T., Olapade-Olaopa E. O., Ogunwobi O. O. (2019). PVT1 signaling is a mediator of cancer progression. *Frontiers in Oncology*.

[38] Martínez-Barriocanal Á., Arango D., Dopeso H. (2020). PVT1 long non-coding RNA in gastrointestinal cancer. *Frontiers in Oncology*.

[39] Wang W., Zhou R., Wu Y. (2019). PVT1 promotes cancer progression via microRNAs. *Frontiers in Oncology*.

[40] Zhou D. D., Liu X. F., Lu C. W., Pant O. P., Liu X. D. (2017). Long non-coding RNA PVT1: emerging biomarker in digestive system cancer. *Cell Proliferation*.

[41] Shen S. N., Li K., Liu Y., Yang C. L., He C. Y., Wang H. R. (2019). Down-regulation of long noncoding RNA PVT1 inhibits esophageal carcinoma cell migration and invasion and promotes cell apoptosis via microRNA-145-mediated inhibition of FSCN1. *Molecular Oncology*.

[42] Shang A. Q., Wang W. W., Yang Y. B. (2019). Knockdown of long noncoding RNA PVT1 suppresses cell proliferation and invasion of colorectal cancer via upregulation of microRNA-214-3p. *American Journal of Physiology. Gastrointestinal and Liver Physiology*.

[43] Barth D. A., Drula R., Ott L. (2020). Circulating non-coding RNAs in renal cell carcinoma-pathogenesis and potential implications as clinical biomarkers. *Frontiers in Cell and Developmental Biology*.

[44] Jiang M.-C., Ni J.-J., Cui W.-Y., Wang B.-Y., Zhuo W. (2019). Emerging roles of lncRNA in cancer and therapeutic opportunities. *American Journal of Cancer Research*.

[45] Kazimierczyk M., Kasprowicz M. K., Kasprzyk M. E., Wrzesinski J. (2020). Human long noncoding RNA interactome: detection, characterization and function. *International Journal of Molecular Sciences*.

[46] Wu H., Wei M., Jiang X. (2020). lncRNA PVT1 promotes tumorigenesis of colorectal cancer by stabilizing miR-16-5p and interacting with the VEGFA/VEGFR1/AKT axis. *Molecular Therapy - Nucleic Acids*.

[47] Bhattacharya R., Fan F., Wang R. (2017). Intracrine VEGF signalling mediates colorectal cancer cell migration and invasion. *British Journal of Cancer*.

