# Supplementary material for: Exosomal lncRNA PVT1/VEGFA Axis Promotes Colon Cancer Metastasis and Stemness by Downregulation of Tumor Suppressor miR-152-3p
Source: Oxid Med Cell Longev. 2021 Jul 15;2021:9959807. doi: 10.1155/2021/9959807 (PMC8315867; doi:10.1155/2021/9959807)
Supplement: Supplementary Materials — Supplementary Table 1: primer sequences used in this study. Supplementary Table 2: clinical table (GSE17537) of Figure 1(b). Supplementary Figure S1: cell viability of different cancer cell lines after exosome treatment. Supplementary Figure S2: gene expression of the PVT1 and VEGF family in different cancer cell lines. Supplementary Figure S3: relative gene expression of miR-152-3p and PVT1 in primary stages. Supplementary Figure S4: full-size blots of Figure 2(a). Supplementary Figure S5: full-size blots of Figure 2(e). Supplementary Figure S6: full-size blots of Figure 4(b). Supplementary Figure S7: full-size blots of Figure 4(e). Supplementary Figure S8: full-size blots of Figure 5(e). [file 9959807.f1.zip › Supplementary data HMS final_0522.docx]

**Exosomal lncRNA PVT1/VEGFA axis promotes colon cancer metastasis and stemness by downregulation of tumor suppressor miR-152-3p**

Shiue-Wei Lai^1,2#^, Ming-Yao Chen^3,4#^, Oluwaseun Adebayo Bamodu^5^, Ming-Shou Hsieh^5^, Ting-Yi Huang^5^, Chi-Tai Yeh^5,8^, Wei-Hwa Lee^9^ and Yih-Giun Cherng^10,11*^

1 Division of Hematology-Oncology, Department of Internal Medicine, Tri-Service General Hospital, National Defense Medical Center, Taipei, Taiwan.

2 Department of Internal Medicine, Tri-Service General Hospital Penghu Branch, Penghu, Taiwan.

3 Division of Gastroenterology and Hepatology, Department of Internal Medicine, Shuang Ho Hospital, New Taipei City, Taiwan.

4 Division of Gastroenterology and Hepatology, Department of Internal Medicine, School of Medicine, College of Medicine, Taipei Medical University, Taipei, Taiwan.

5 Department of Medical Research & Education, Taipei Medical University - Shuang Ho Hospital, New Taipei City, 235, Taiwan.

6 Department of Medical Laboratory Science and Biotechnology, Yuanpei University of Medical Technology, Hsinchu 300, Taiwan.

7 Department of Pathology, Taipei Medical University-Shuang Ho Hospital, New Taipei City, Taiwan

8 Department of Anesthesiology, Shuang Ho Hospital, Taipei Medical University, New Taipei City, Taiwan.

9 Department of Anesthesiology, School of Medicine, College of Medicine, Taipei Medical University, Taipei, Taiwan.

# These authors have contributed equally to this work

* Correspondence:

Dr. [Yih-Giun Cherng](https://pubmed.ncbi.nlm.nih.gov/?term=Cherng+YG&cauthor_id=29329323), MD

Department of Anesthesiology, Shuang Ho Hospital, Taipei Medical University, New Taipei City, Taiwan. No. 291, Zhongzheng Road, Zhonghe District, New Taipei City, Taiwan 235.

Email: [stainless@s.tmu.edu.tw](mailto:stainless@s.tmu.edu.tw)

Tel.: +886-2-2490088 ext. 8885

**Supplementary Table 1.** Primer sequences used in this study

| Gene | Forward sequence | Reverse sequence |
| --- | --- | --- |
| *PVT1* | TCGAGGTCATAGTTCCTGTTGG | GCCCCTTCTATGGGAATCACTA |
| *EGFR* | GGCACTTTTGAAGATCATTTTCTC | CTGTGTTGAGGGCAATGAG |
| *VEGFA* | ATCTGCATGGTGATGTTGGA | GGGCAGAATCATCACGAAGT |
| *GAPDH* | GAGTCAACGGATTTGTCGT | GACAAGCTTCCCGTTCTCAG |
| *RPL19* | ATGACTGCATCGTTGATAAAATCC | GGCGCAAAATCCTCATTCTC |

**Supplementary Table 2.** Clinical table (GSE17537) of Figure 1B

**
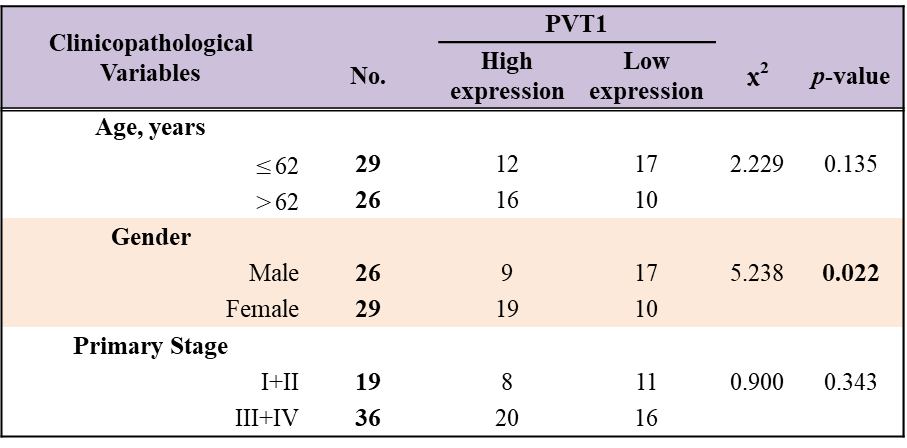
**

**Supplementary Figure S1.** Cell viability of different cancer cell lines with exosome treatment.

**Supplementary Figure S2.** Gene expression of PVT1 and VEGF family in different cancer cell lines.

**Supplementary Figure S3.** Relative gene expression of miR-152-3p and PVT1 in primary stages


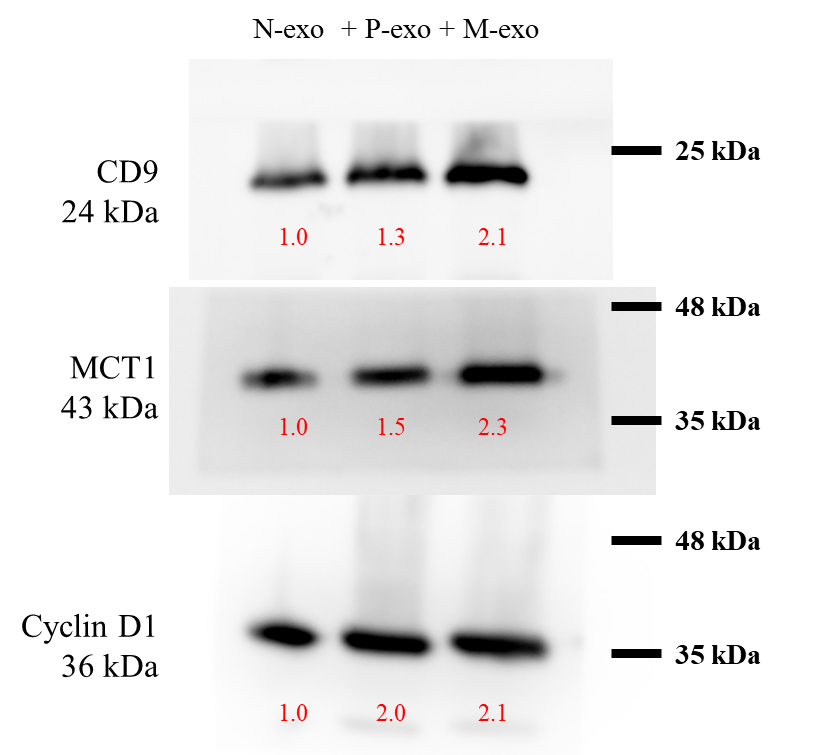


**Supplementary Figure S4.** Full-size blots of Figure 2A


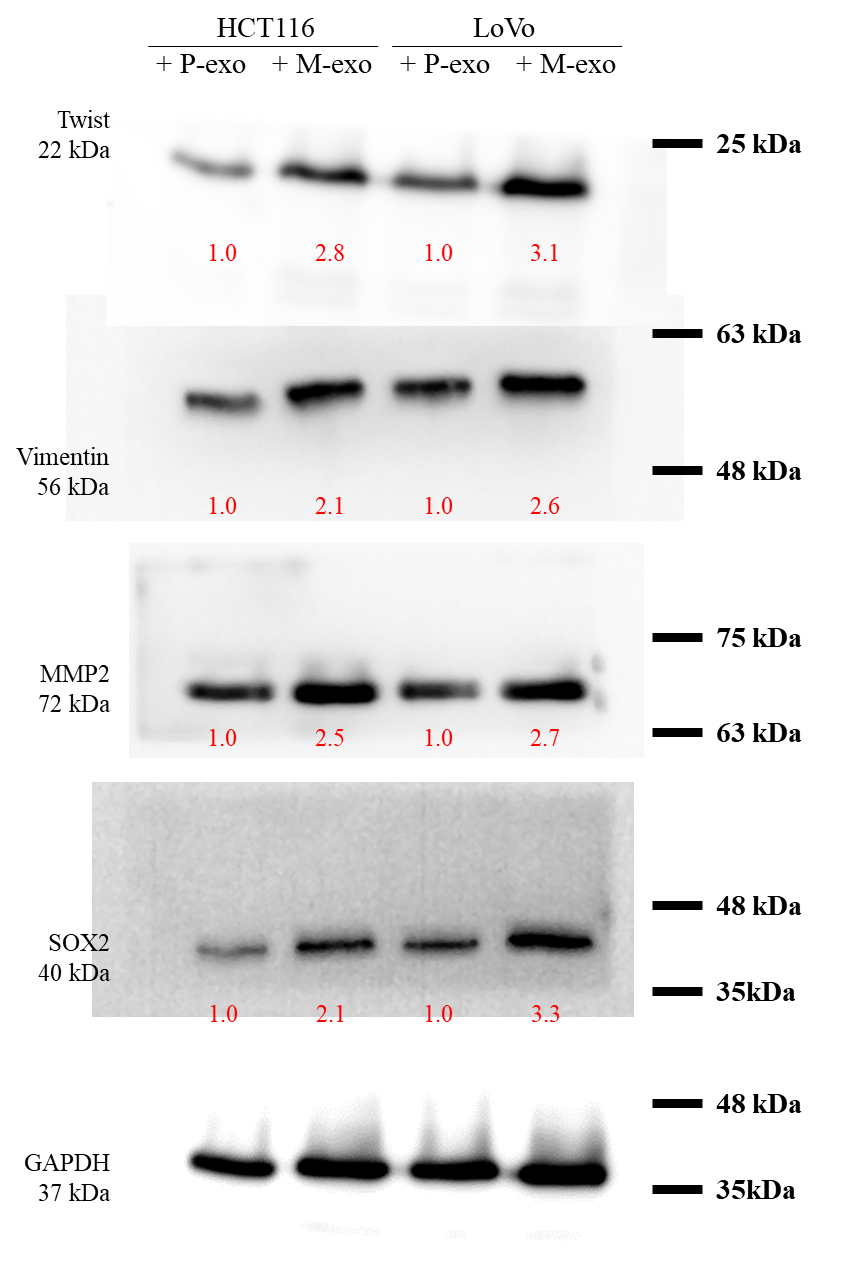


**Supplementary Figure S5.** Full-size blots of Figure 2E


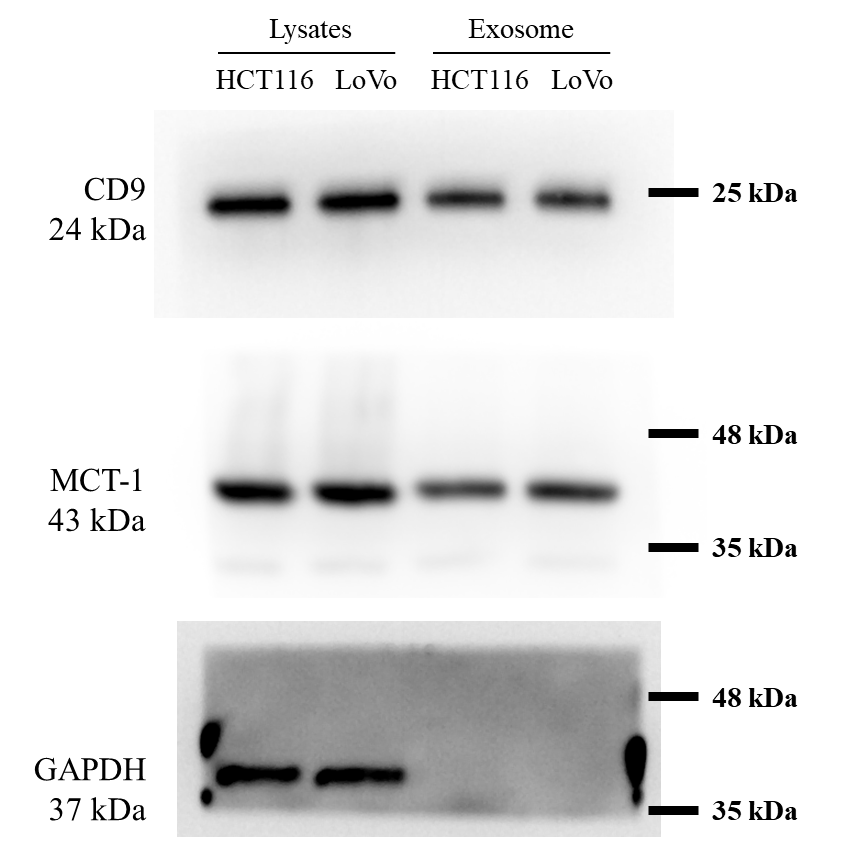


1.0

1.0

0.8

0.7

1.1

1.0

0.8

1.0

0.9

1.0

**Supplementary Figure S6.** Full-size blots of Figure 4B

**
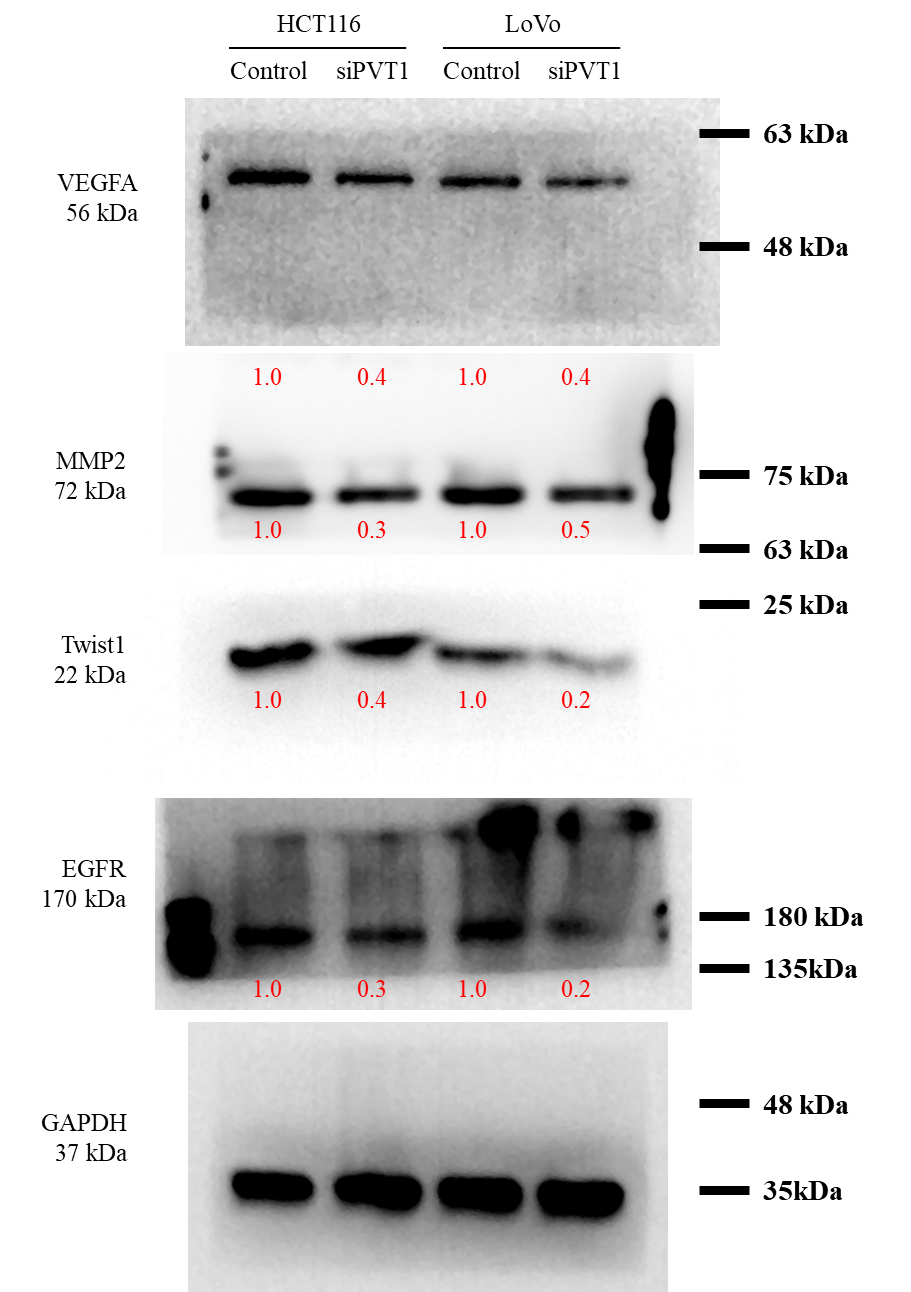
**

**Supplementary Figure S7.** Full-size blots of Figure 4E


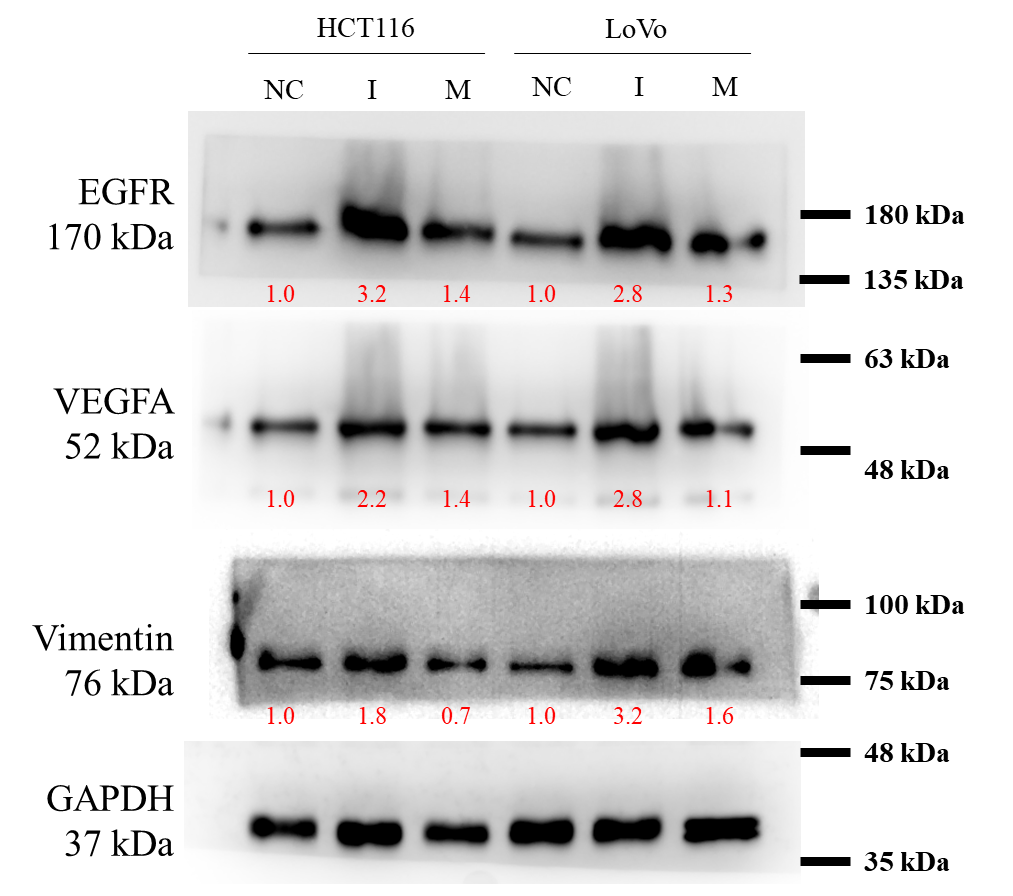


**Supplementary Figure S8.** Full-size blots of Figure 5E
