# Supplementary material for: Exosomal lncRNA PVT1/VEGFA Axis Promotes Colon Cancer Metastasis and Stemness by Downregulation of Tumor Suppressor miR-152-3p
Source: Oxid Med Cell Longev. 2021 Jul 15;2021:9959807. doi: 10.1155/2021/9959807 (PMC8315867; doi:10.1155/2021/9959807)
Supplement: Supplementary Materials — Supplementary Table 1: primer sequences used in this study. Supplementary Table 2: clinical table (GSE17537) of Figure 1(b). Supplementary Figure S1: cell viability of different cancer cell lines after exosome treatment. Supplementary Figure S2: gene expression of the PVT1 and VEGF family in different cancer cell lines. Supplementary Figure S3: relative gene expression of miR-152-3p and PVT1 in primary stages. Supplementary Figure S4: full-size blots of Figure 2(a). Supplementary Figure S5: full-size blots of Figure 2(e). Supplementary Figure S6: full-size blots of Figure 4(b). Supplementary Figure S7: full-size blots of Figure 4(e). Supplementary Figure S8: full-size blots of Figure 5(e). [file 9959807.f1.zip › Supplementary data HMS final_0522-Meraged.pdf]

Exosomal lncRNA PVT1/VEGFA axis promotes colon cancer metastasis and stemness by downregulation of tumor suppressor miR-152-3p

Shiue-Wei Lai[1,2#], Ming-Yao Chen[3,4#], Oluwaseun Adebayo Bamodu[5], Ming-Shou Hsieh[5], Ting-Yi Huang[5], Chi-Tai Yeh[5,8], Wei-Hwa Lee[9] and Yih-Giun Cherng[10,11*]

1 Division of Hematology-Oncology, Department of Internal Medicine, Tri-Service General Hospital, National Defense Medical Center, Taipei, Taiwan.

2 Department of Internal Medicine, Tri-Service General Hospital Penghu Branch, Penghu, Taiwan.

3 Division of Gastroenterology and Hepatology, Department of Internal Medicine, Shuang Ho Hospital, New Taipei City, Taiwan.

4 Division of Gastroenterology and Hepatology, Department of Internal Medicine, School of Medicine, College of Medicine, Taipei Medical University, Taipei, Taiwan.

5 Department of Medical Research & Education, Taipei Medical University - Shuang Ho Hospital, New Taipei City, 235, Taiwan.

6 Department of Medical Laboratory Science and Biotechnology, Yuanpei University of Medical Technology, Hsinchu 300, Taiwan.

7 Department of Pathology, Taipei Medical University-Shuang Ho Hospital, New Taipei City, Taiwan

8 Department of Anesthesiology, Shuang Ho Hospital, Taipei Medical University, New Taipei City, Taiwan.

9 Department of Anesthesiology, School of Medicine, College of Medicine, Taipei Medical University, Taipei, Taiwan.

# These authors have contributed equally to this work

* Correspondence:

Dr. Yih-Giun Cherng, MD

Department of Anesthesiology, Shuang Ho Hospital, Taipei Medical University, New Taipei City, Taiwan. No. 291, Zhongzheng Road, Zhonghe District, New Taipei City, Taiwan 235.

Email: stainless@s.tmu.edu.tw

Tel.: +886-2-2490088 ext. 8885

Supplementary Table 1. Primer sequences used in this study

Gene	Forward sequence	Reverse sequence
PVT1	TCGAGGTCATAGTTCCTGTTGG	GCCCCTTCTATGGGAATCACTA
EGFR	GGCACTTTTGAAGATCATTTTCTC	CTGTGTTGAGGGCAATGAG
VEGFA	ATCTGCATGGTGATGTTGGA	GGGCAGAATCATCACGAAGT
GAPDH	GAGTCAACGGATTTGTCGT	GACAAGCTTCCCGTTCTCAG
RPL19	ATGACTGCATCGTTGATAAAATCC	GGCGCAAAATCCTCATTCTC

Supplementary Table 2. Clinical table (GSE17537) of Figure 1B

Clinicopathological Variables	No.	PVT1		x^2	*p*-value
		High expression	Low expression		
**Age, years**					
≤62	**29**	12	17	2.229	0.135
>62	**26**	16	10		
**Gender**					
Male	**26**	9	17	5.238	**0.022**
Female	**29**	19	10		
**Primary Stage**					
I+II	**19**	8	11	0.900	0.343
III+IV	**36**	20	16		

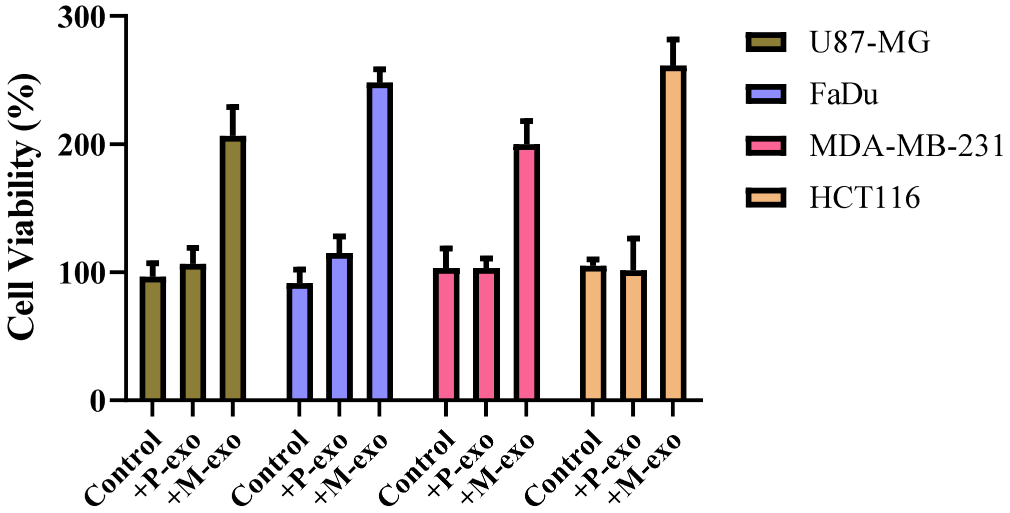

Supplementary Figure S1. Cell viability of different cancer cell lines with exosome treatment.

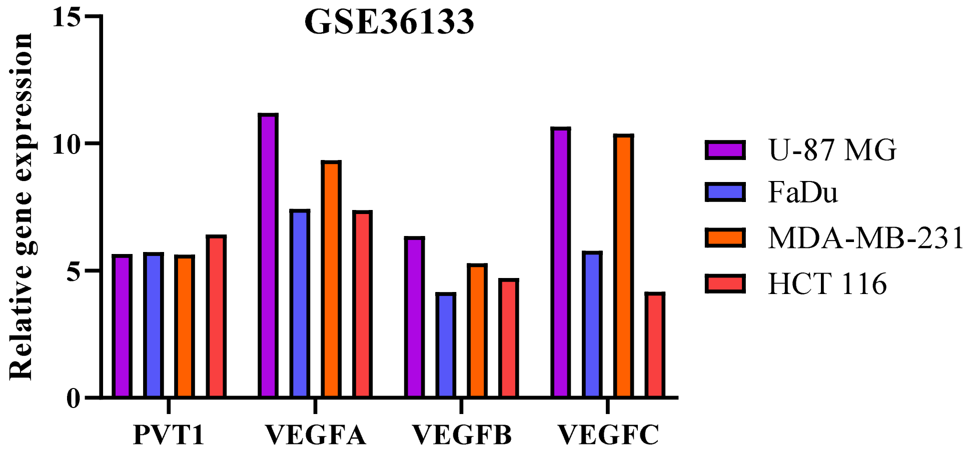

Supplementary Figure S2. Gene expression of PVT1 and VEGF family in different cancer cell lines.

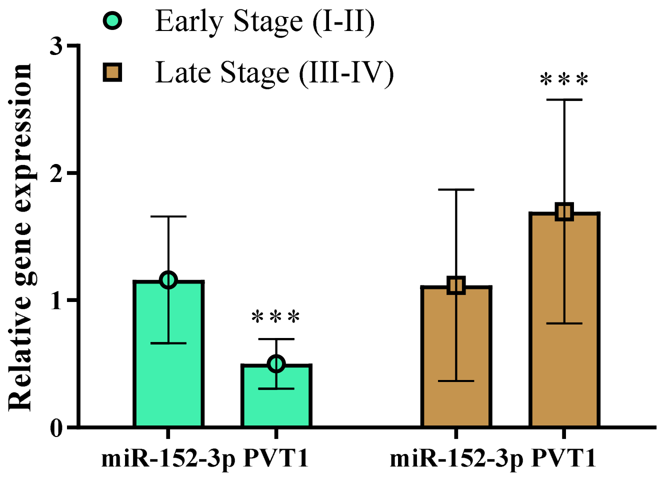

Supplementary Figure S3. Relative gene expression of miR-152-3p and PVT1 in primary stages

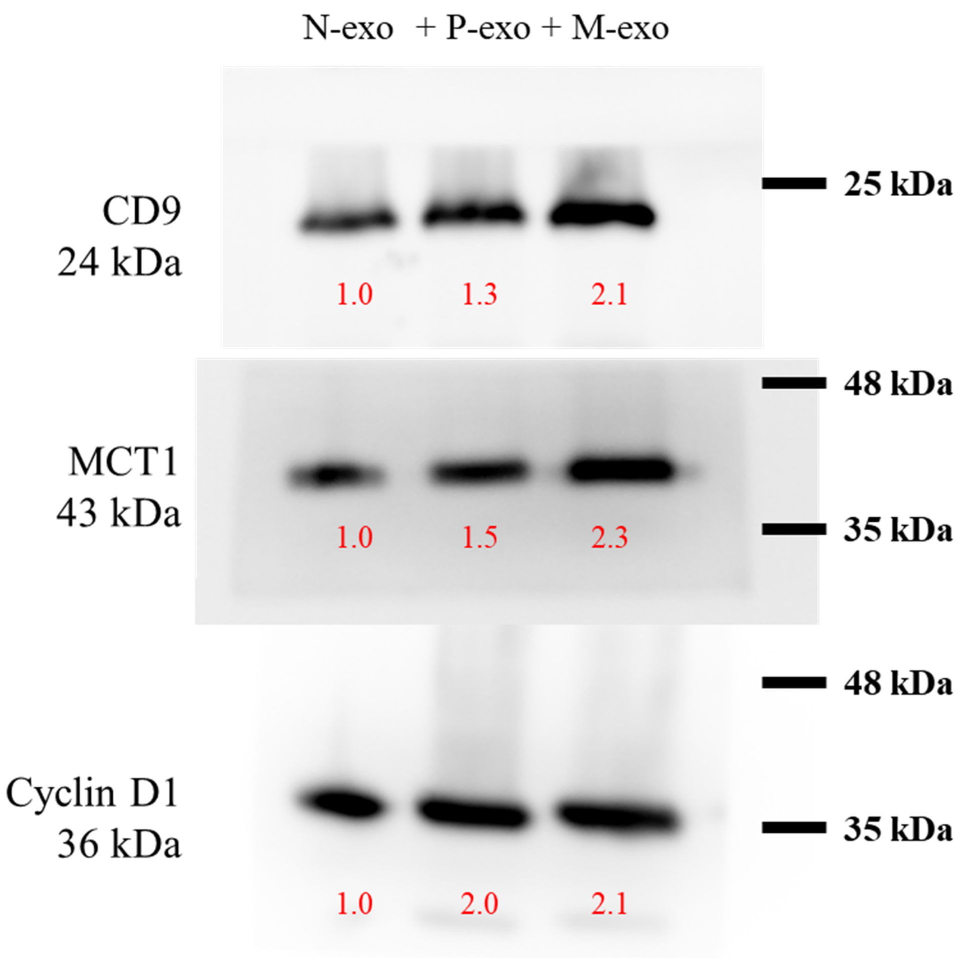

Supplementary Figure S4. Full-size blots of Figure 2A

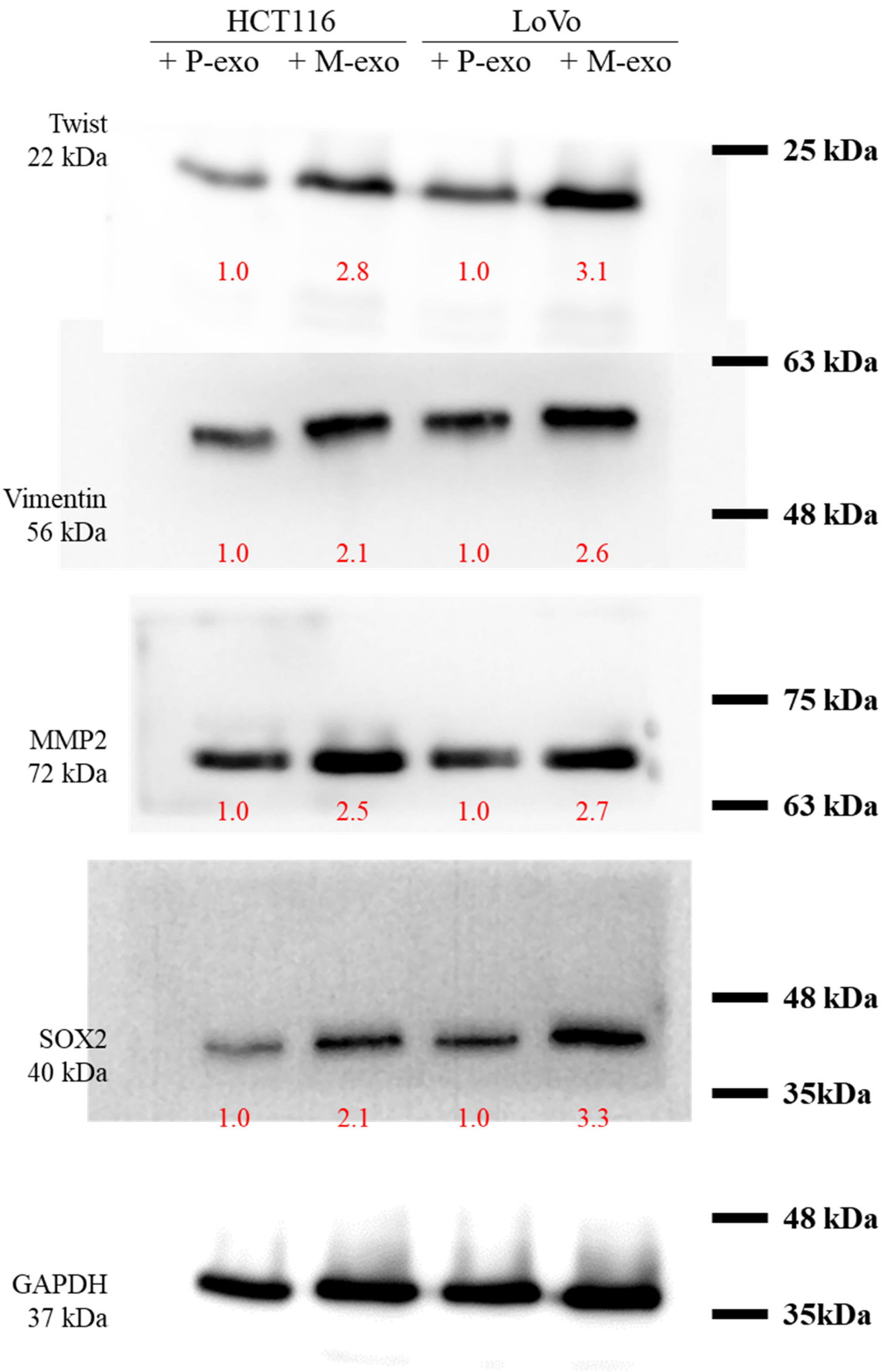

Supplementary Figure S5. Full-size blots of Figure 2E

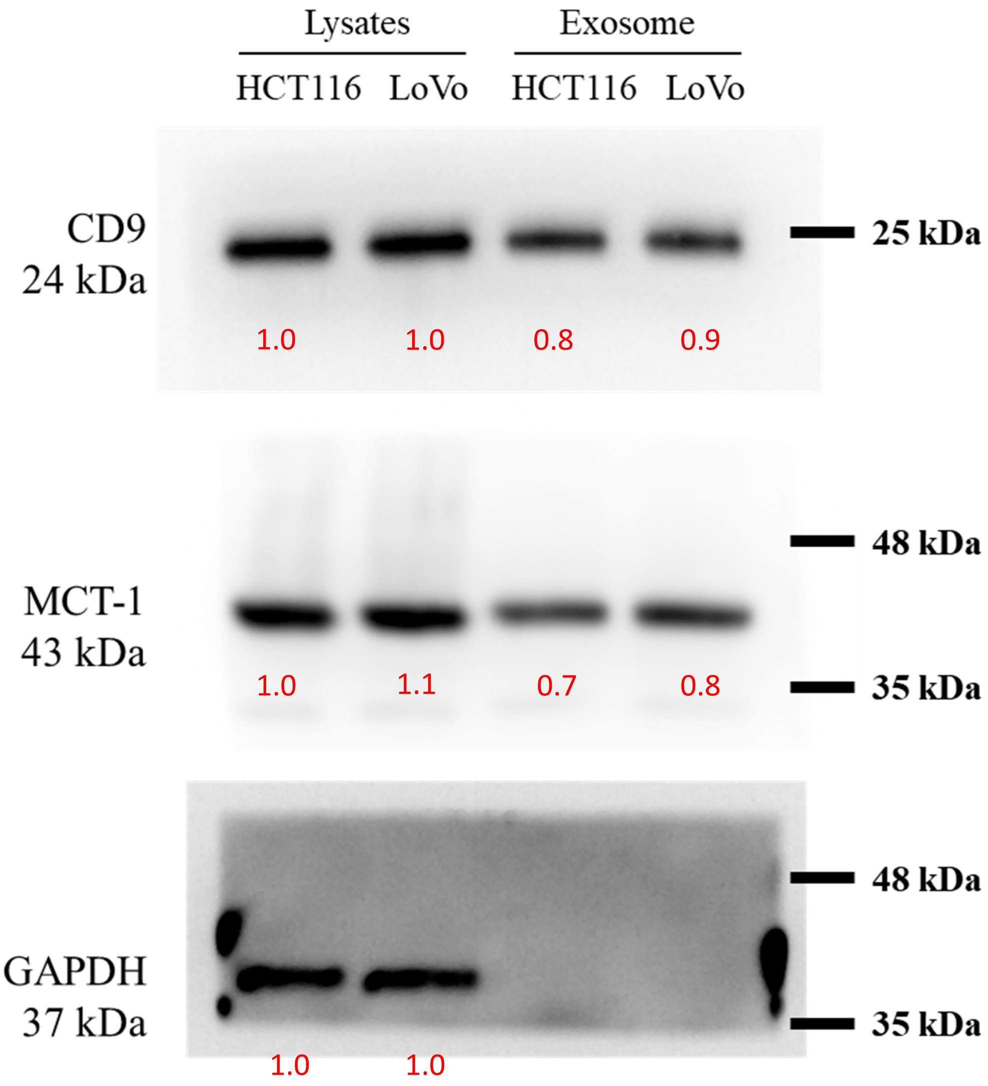

Supplementary Figure S6. Full-size blots of Figure 4B

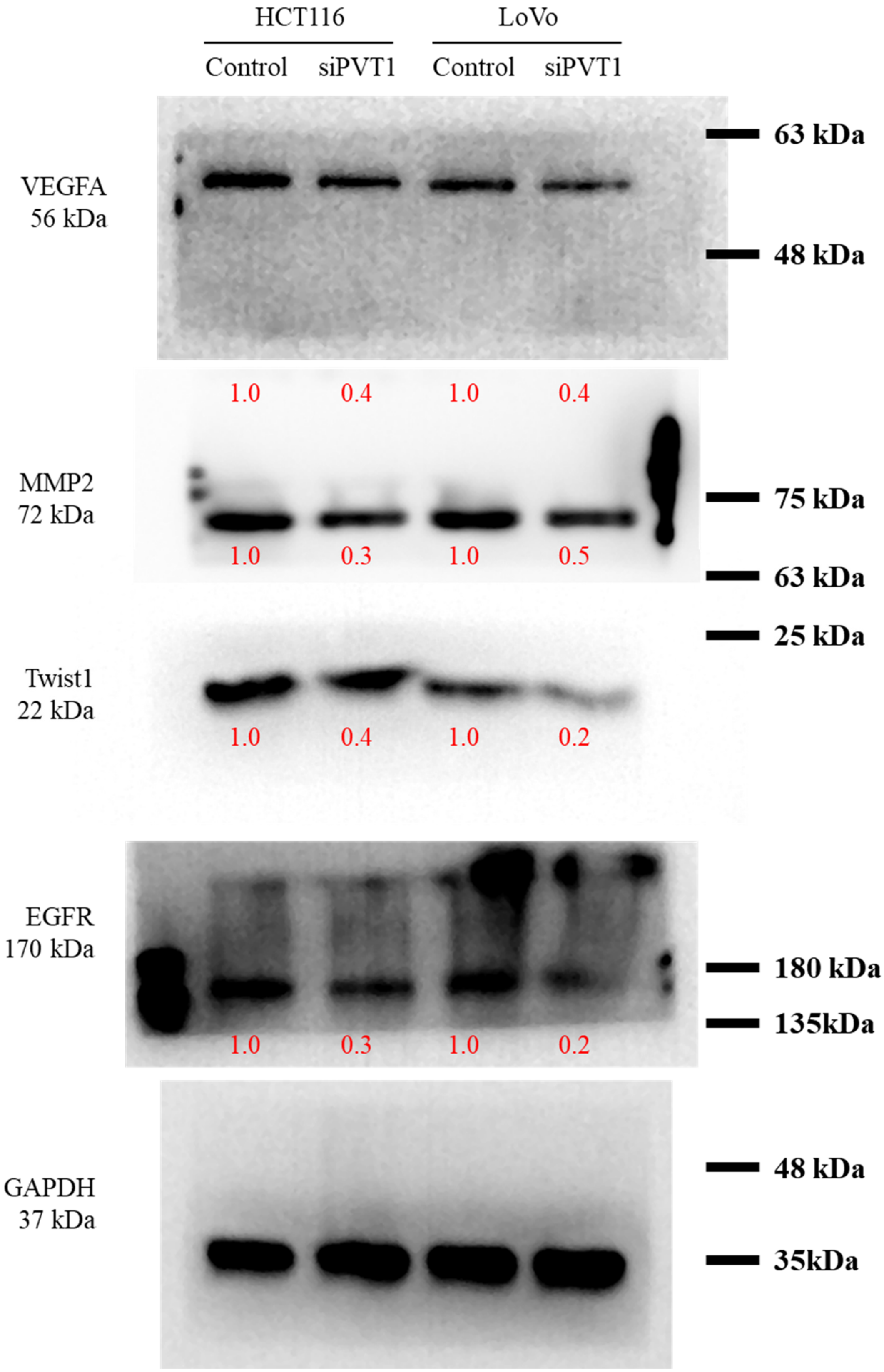

Supplementary Figure S7. Full-size blots of Figure 4E

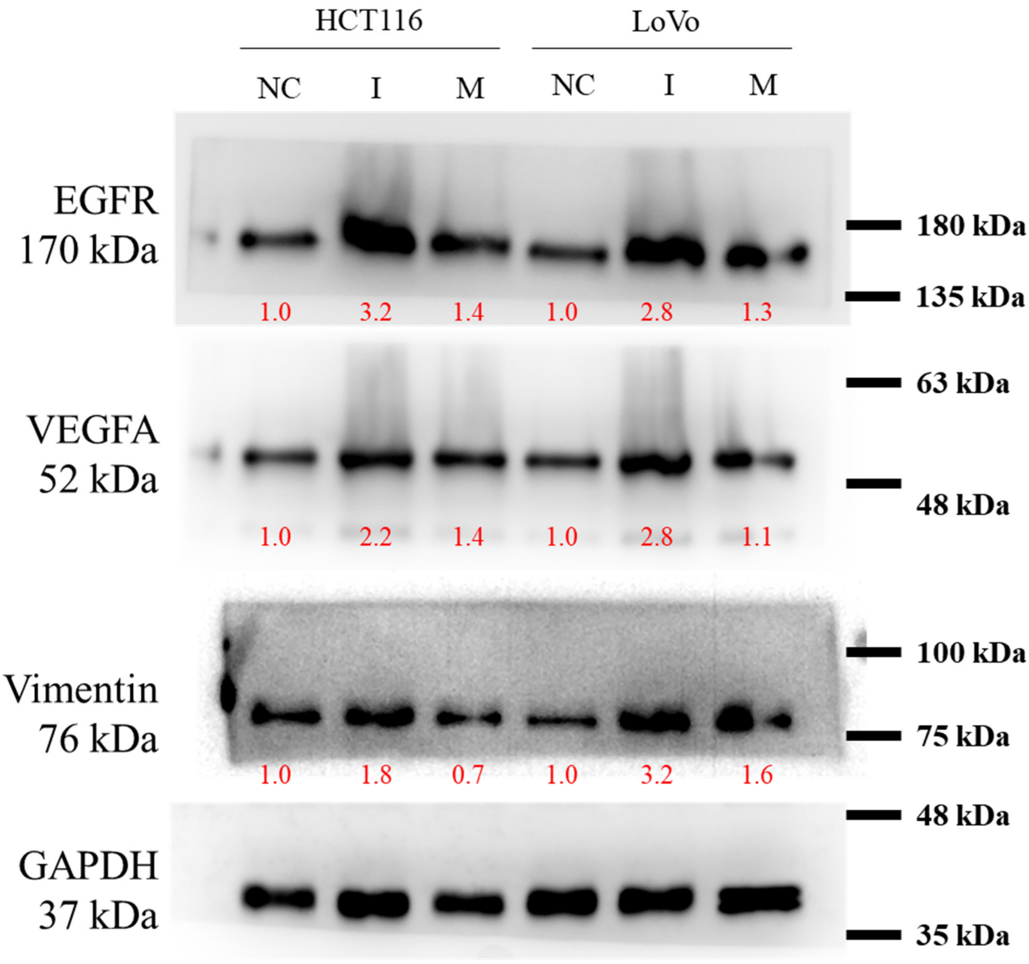

Supplementary Figure S8. Full-size blots of Figure 5E

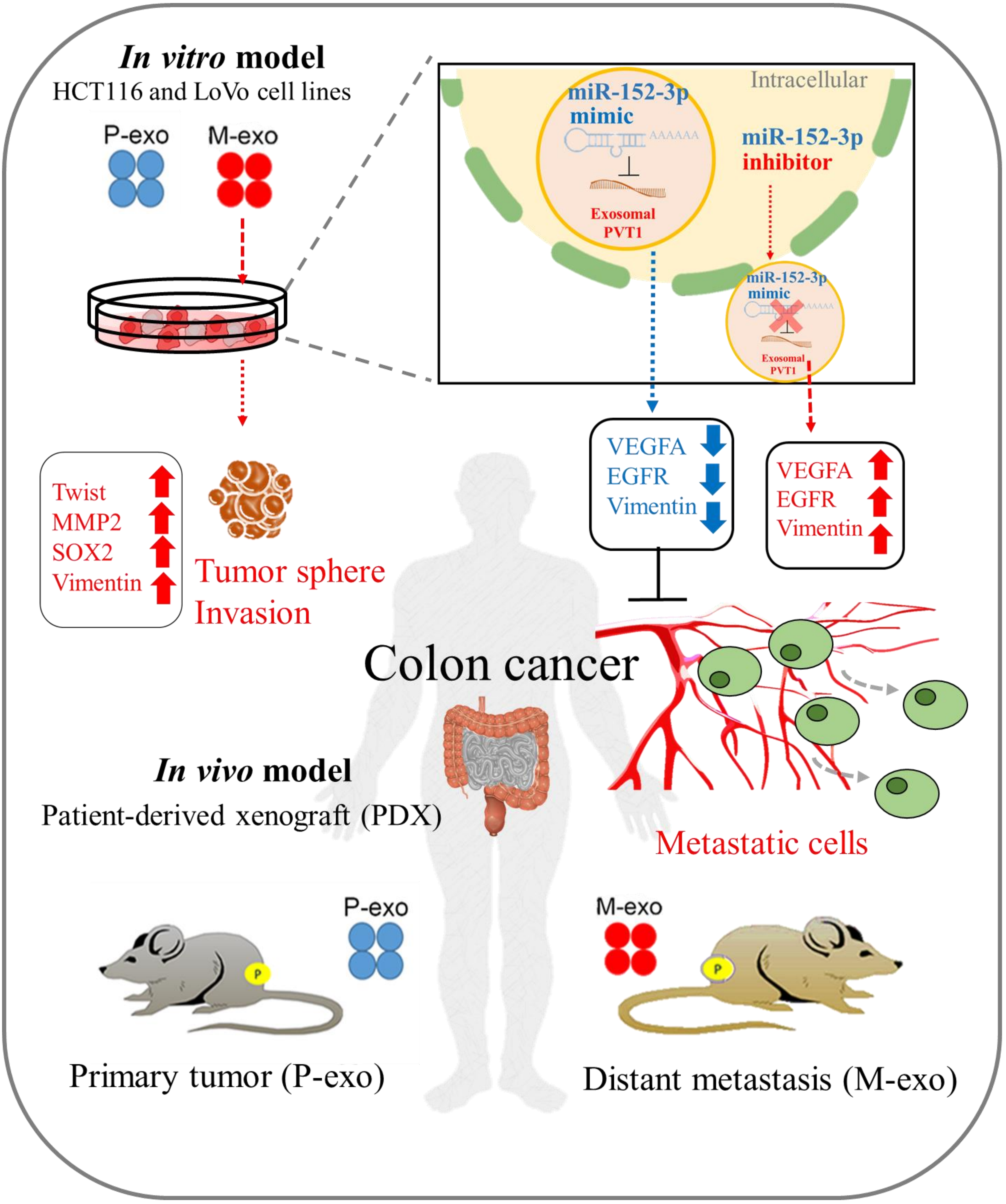

In vitro model
HCT116 and LoVo cell lines
P-exo
M-exo
Intracellular
miR-152-3p mimic
AAAAAA
Exosomal PVT1
miR-152-3p inhibitor
miR-152-3p mimic
Exosomal PVT1
VEGFA
EGFR
Vimentin
VEGFA
EGFR
Vimentin
Twist
MMP2
SOX2
Vimentin
Tumor sphere
Invasion
Colon cancer
In vivo model
Patient-derived xenograft (PDX)
Metastatic cells
P-exo
M-exo
Primary tumor (P-exo)
Distant metastasis (M-exo)

Figure 6
